# Disentangling the Role of Working Memory in Parkinson’s Disease

**DOI:** 10.3389/fnagi.2020.572037

**Published:** 2020-09-25

**Authors:** Juha Salmi, Liisa Ritakallio, Daniel Fellman, Ulla Ellfolk, Juha O. Rinne, Matti Laine

**Affiliations:** ^1^Department of Neuroscience and Biomedical Engineering, Aalto University, Espoo, Finland; ^2^Department of Psychology and Speech-Language Pathology, University of Turku, Turku, Finland; ^3^Turku Institute for Advanced Studies, University of Turku, Turku, Finland; ^4^Department of Psychology, Åbo Akademi University, Turku, Finland; ^5^Department of Applied Educational Science, Umeå University, Umeå, Sweden; ^6^Department of Psychiatry, Visby County Hospital, Visby, Sweden; ^7^Division of Clinical Neurosciences, Turku University Hospital, Turku, Finland; ^8^Turku PET Centre, University of Turku, Turku, Finland; ^9^Turku Brain and Mind Center, University of Turku, Turku, Finland

**Keywords:** Parkinson’s disease, working memory, affective symptoms, depression, cognitive impairment

## Abstract

Working memory (WM) represents a core cognitive function with a major striatal contribution, and thus WM deficits, commonly observed in Parkinson’s disease (PD), could also relate to many other problems in PD patients. Our online study aimed to determine the subdomains of WM that are particularly affected in PD and to clarify the links between WM and everyday cognitive deficits, other executive functions, psychiatric and PD symptoms, as well as early cognitive impairment. Fifty-two mild-to-moderate PD patients and 54 healthy controls performed seven WM tasks tapping selective updating, continuous monitoring, or maintenance of currently active information. Self-ratings of everyday cognition, depression, and apathy symptoms, as well as screenings of global cognitive impairment, were also collected. The data were analyzed using structural equation modeling. Of the three WM domains, only selective updating was directly predictive of PD group membership. More widespread WM deficits were observed only in relation to global cognitive impairment in PD patients. Self-rated everyday cognition or psychiatric symptoms were not linked to WM performance but correlated with each other. Our findings suggest that WM has a rather limited role in the clinical manifestation of PD. Nevertheless, due to its elementary link to striatal function, the updating component of WM could be a candidate for a cognitive marker of PD also in patients who are otherwise cognitively well-preserved.

## Introduction

Parkinson’s disease (PD) is characterized by complex symptomatology including not only motor symptoms but also a decline of cognitive function and psychiatric well-being (Kalia and Lang, 2015). PD is associated with progressive death of dopaminergic neurons in the substantia nigra that disrupts outflow in the cortico-striatal circuitries (Owen et al., 1998) and results in lowered dopamine levels (Owen et al., 1997). The role of the nigro-striatal and cortico-striatal circuitries in PD patients’ difficulties in motor coordination and timing has been known for a century (Tretiakoff, 1919). Later it has been established that especially the dorsal striatum also contributes to the regulation of cognitive functions (Shepherd, 2013), giving rise to at least some of the cognitive deficits in PD.

In healthy adults, working memory (WM) is currently amongst the most extensively studied cognitive functions related to the dorsal striatum (e.g., Frank et al., 2001; Cools and D’Esposito, 2011; Bäckman and Nyberg, 2013). WM allows us to maintain and manipulate information in mind, monitor the ongoing situation, and update incoming information to refresh the currently active memory contents (Eriksson et al., 2015). Due to the striatal dysregulation in PD, this disorder has been considered as a model of WM dysfunction already decades ago (Goldman-Rakic, 1995; see also Gotham et al., 1988; Levin et al., 1989). Later neurobiological and brain imaging studies in healthy adults have concluded that WM typically engages widespread frontoparietal networks (Rottschy et al., 2012; Eriksson et al., 2015), although some evidence indicates that especially the cortico-striatal loops are specifically linked to updating of information in WM (Frank et al., 2001; O’Reilly and Frank, 2006). This raises the question of whether WM deficits in PD concern particularly this subdomain. Despite the extensive evidence of WM deficits in PD (e.g., Bradley et al., 1989; Owen et al., 1997; Altgassen et al., 2007; Siegert et al., 2008; Lee et al., 2010), the susceptibility of different WM subdomains to PD has not been examined in detail. Another important issue is whether deficits in such a central cognitive function as WM and its subdomains (e.g., D’Esposito and Postle, 2015) are related to other cognitive as well such as psychiatric deficits, potentially hampering everyday lives of the patients (e.g., Hofmann et al., 2012; Alloway and Horton, 2016). Several studies have demonstrated that particularly in PD patients, depression/anxiety is associated with abnormal functioning of the cortico-striatal loops (Remy et al., 2005; Joutsa et al., 2013; Qian et al., 2017). Thus, a link between WM and psychiatric symptoms could be expected because of the overlapping neuropathology (Joutsa et al., 2013).

The dysregulation of the frontostriatal circuit is not the only pathological feature in PD. The disease also results in abnormal aggregates of alpha-synuclein protein, Lewy bodies, and Lewy neurites during the disease (Spillantini et al., 1998). These distributed neuropathologies are associated with common symptomatologies in dementing processes such as global cognitive decline (Emre et al., 2007; Santangelo et al., 2014) and geriatric depression (Zgaljardic et al., 2003). Moreover, there are also other cognitive domains besides WM that are affected in dementing disorders including PD. These include executive function, attention, and inhibition of irrelevant information or inappropriate behaviors (Emre, 2003; Litvan et al., 2012; Koster et al., 2015). In Alzheimer’s disease, there is evidence that managing everyday situations is associated not only with objectively measured cognitive abilities but also with psychiatric symptoms (Balash et al., 2013). However, in PD, the corresponding links remain unclear.

In this study, we examined the role of WM in PD in more detail than has been done in previous studies. Based on earlier research, our WM tasks were chosen to tap into the following hypothetical WM subdomains: selective updating, maintenance of information, and continuous monitoring. Updating and maintenance of information are often separated in WM research (e.g., Nyberg and Eriksson, 2016), and this division is also supported by some factor-analytic studies (e.g., Schmiedek et al., 2009). Inclusion of WM updating tasks was directly based on our hypothesis that difficulties in especially this WM subdomain (Frank et al., 2001; Cools and D’Esposito, 2011; Bäckman and Nyberg, 2013) would be observed among the PD patients due to the critical role the frontostriatal system in updating performance (Owen et al., 1998). Maintenance of information in WM, in turn, is another subdomain commonly affected in aging-related disorders (see Reuter-Lorenz and Sylvester, 2005). Continuous monitoring has been suggested as a possibly distinct WM subdomain only more recently (Waris et al., 2017). A confirmatory factor analysis was conducted to test this division into three WM subdomains [see Supplementary Online Methods (SOM), Supplementary Tables S1, S2]. In selecting the WM tasks, we also considered the reliability of the measures (see Soveri et al., 2007, and the “Materials and Methods” section). As regards other cognitive domains, we compiled a battery measuring attention and inhibition, processing speed, resistance to interference, and episodic memory. These tasks were selected to cover cognitive domains other than WM that have often shown impairments in PD (Levin et al., 1989; Zgaljardic et al., 2003; Kandiah et al., 2009; Biundo et al., 2016; Aarsland et al., 2017). Besides these objective measures of cognitive abilities, we assessed self-experienced management of everyday situations demanding executive function and WM, as well as self-assessed apathy and depression symptoms. Global cognitive abilities potentially reflecting early dementia symptoms as well as non-motor PD symptoms were assessed with telephone interviews and self-ratings. The links between these various measures in PD vs. healthy aged participants were then examined using structural equation models (SEM). To further investigate the association between WM performance and global cognitive abilities or PD symptoms, we performed simple linear regression analyses within the PD group.

Despite cognitive deficits at the group-level, it is worth underscoring that many PD patients remain cognitively rather intact even in the presence of severe PD symptoms or psychiatric issues (Aarsland et al., 2017). Also in those PD patients who do show a cognitive decline, severity can vary from PD mild cognitive impairment (PD-MCI; Litvan et al., 2012) to PD dementia (PD-D; Emre et al., 2007). It has also been suggested that PD patients can sometimes experience everyday cognitive deficits even when there is no decline in objective cognitive task performance (subjective cognitive decline; Erro et al., 2014; Aarsland et al., 2017). As is typical for dementing disorders in general (Hendrie et al., 1997), affective symptoms and cognitive dysfunction frequently co-occur in PD (Weintraub and Stern, 2005; de la Riva et al., 2014). Indeed, psychiatric symptoms are amongst the most significant predictors of cognitive decline in PD (Aarsland et al., 2003; Hobson and Meara, 2004; Uc et al., 2009; Kwon et al., 2014; Chen et al., 2016). It is, however, unclear whether, and how the psychiatric symptoms and impaired global cognitive abilities specifically relate to WM deficits.

Based on previous studies, we put forth the following hypotheses. First, due to the well-established role of the cortico-striatal loops in WM updating (Frank et al., 2001; Cools and D’Esposito, 2011; Bäckman and Nyberg, 2013), we hypothesized that this WM subdomain would show WM deficits even in PD patients with mild-to-moderate symptoms. As there is evidence of problems of WM functioning also in other WM subdomains in PD patients, we expected that also other WM deficits could be observed, especially if the global cognitive functioning was affected (Siegert et al., 2008). Second, based on the role of the cortico-striatal loops in depression/anxiety (Remy et al., 2005; Joutsa et al., 2013; Qian et al., 2017) and prior behavioral evidence (Aarsland et al., 2003; Hobson and Meara, 2004; Uc et al., 2009; Chen et al., 2016), we expected to find a link between WM and depression/anxiety symptoms commonly observed in PD. Or perhaps, the patients’ self-experienced everyday difficulties in cognitively demanding situations could rather reflect psychiatric factors. This would be supported by prior behavioral evidence on SCD (Erro et al., 2014; Aarsland et al., 2017).

For the data collection, we administered a full-blown Internet-based assessment that is becoming increasingly common when collecting neuropsychological testing data. We have successfully used these methods in collecting large-scale data on WM performance (see Waris et al., 2017; Laine et al., 2018), including patients with PD (Fellman et al., 2020). The reliability of the online testing in this PD sample was more thoroughly analyzed in our prior study (Fellman et al., 2020; see also Mackin et al., 2018; Weil et al., 2018).

## Materials and Methods

### Participants

Altogether 52 PD patients and 54 healthy controls completed the study. The PD patients were recruited *via* the Finnish Parkinson Association website, seminars, and brochures distributed at various events. The healthy controls were mainly recruited *via* the SeniorSurf network^1^. We attempted to match the control participants to the PD participants at the group level in terms of age and education range, gender ratio, distribution of work situation, and geographical location (see Table 1). The study was conducted following the Helsinki Declaration and it was approved by the Ethics Committee of the University of the Hospital District of Southwest Finland. Informed consent was obtained from all participants before enrolment. See SOM for details.

**Table 1:** Demographic and clinical characteristics of the sample.

Measure	Dependent variable	PD patients (*n* = 52)	Healthy controls (*n* = 54)	*p*	Cohen’s *d*
**Demographics**				
Age	Years	65.12 (5.46)	65.96 (4.10)	0.71^b^	0.17
Gender	Female/male	34/18	41/13	0.29^c^	n/a
Education	Years	14.79 (4.75)	14.22 (3.85)	0.64^b^	−0.13
**Clinical characteristics**				
Age at diagnosis	Years	59.5 (7.2)	n/a		
Disease duration	Years	5.6 (4.8)	n/a		
Levodopa equivalent daily dose	mg/day	458.27 (362.52)	n/a		
UPDRS part I	Sum score	3.12 (2.54)	n/a		
UPDRS part II	Sum score	9.31 (5.29)	n/a		
PDQ	Sum score	17.28 (11.73)	n/a		
SPDDS	Sum score	31.48 (6.85)	n/a		
**Global cognition**					
TELE	Sum score	19.62 (0.63)	19.62 (0.52)	0.67^b^	0
TICS-m	Sum score	37.17 (3.13)	39.44 (3.54)	<0.01^b^	0.68
**Self-reported cognitive symptoms**					
WMQ	Sum score	57.85 (16.57)	48.85 (12.57)	<0.01^b^	−0.61
BRIEF-A	Sum score	48.04 (17.13)	40.00 (18.65)	0.02^b^	−0.45
**Self-reported affective symptoms**					
GDS-30	Sum score	6.19 (5.40)	2.98 (3.84)	<0.01^b^	−0.69
LARS	Sum score	−27.10 (5.02)	−28.06 (3.71)	0.50^b^	−0.22
**Motivation**					
Motivation throughout the test period	Mean	4.12 (0.69)	4.04 (0.73)	0.57^a^	−0.11
**Alertness**					
Alertness throughout the test period	Mean	3.58 (0.79)	3.66 (0.69)	0.60^a^	0.10

*Note. Values in parentheses are standard deviations. UPDRS, Unified Parkinson Disease Rating Scale; PDQ-39, Parkinson’s Disease Questionnaire-39; SPDDS, Self-assessment Parkinson’s Disease Disability Scale; TELE, Telephone Screening Protocol; TICS-m, Telephone Interview for Cognitive Status-modified; WMQ, Working Memory Questionnaire; BRIEF-A, Behavior Rating Inventory of Executive Function; GDS-30, Geriatric Depression Scale-30; LARS, Lille Apathy Rating Scale. ^a^Independent samples *t*-test. ^b^Mann–Whitney’s U-test. ^c^Fisher’s exact test, two-tailed*.

### Design and Procedure

**Figure 1:**
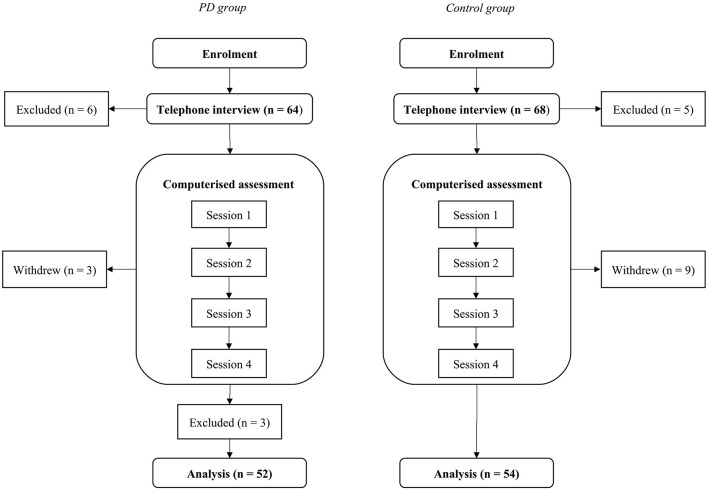
Chart for the study procedure. The Parkinson’s disease (PD) group is shown on the left and the control group on the right.

The data collection was divided into two main phases over 2 weeks. The first phase was a telephone interview, in which eligibility for the computerized phase was assessed. The second phase was an Internet-based computerized task and questionnaire battery. Thus, all data collection was done on the telephone and online, either on the participant’s home computer or on the computer of their family or friends. The participants had an opportunity to receive assistance whenever they needed it. See Figure 1 for the procedure and SOM for some details.

For the PD participants, the duration of the telephone interview that also included PD symptom screening was approximately 90 min. For the control group participants, the duration of the telephone interview was approximately 30 min. The participants were prescreened for the following exclusion criteria: dyslexia, neurological disorders other than PD (including traumatic brain injury and stroke), psychiatric disorders, severe motor fluctuations and involuntary muscle movements (i.e., dyskinesias), exposure to two or more languages before the age of six, and other medications than those related to PD that would affect the CNS. Additionally, they were asked about any other health issues that might pose problems for participation, either by significantly affecting the results (e.g., impaired perception of stimuli) or their overall well-being during the study. Six participants in the PD group and five healthy controls were excluded for meeting one or more of the aforementioned exclusion criteria. In the PD group, two participants were excluded for bilingualism, three for other neurological disorders, and one for other severe health issues. In the control group, one participant was excluded for bilingualism, two for neurological disorders, one for CNS medication, and one for other severe health issues.

The remaining PD and control participants were further prescreened for global cognitive impairment with the Telephone Interview for Cognitive Status-modified (TICS-m; Welsh et al., 1993) and with the Telephone Screening Protocol (TELE; Gatz et al., 1995). No one was excluded at this point, as all scored above the dementia cut-off for both measures. The remaining PD participants were also interviewed regarding the nature and severity of their PD symptoms with the Unified Parkinson’s Disease Rating Scale (UPDRS; Fahn and Elton, 1987) parts I and II. Additionally, we asked if they considered their motor or other symptoms to be such that the computerized phase of the study would cause them difficulty or distress. No participant considered their symptoms to prevent them from proceeding to the computerized phase. The interview ended with instructions regarding the study setup, specifically the schedule and technical aspects of the online testing phase.

At the online testing phase, the participants received an e-mail containing detailed instructions as to how to conduct this part of the experiment (e.g., to set up a place where there are no distractors or interruptions), and a link to the test platform. They then proceeded to the online testing independently, although, in case of technical and other issues, they were able to contact the researchers by e-mail or telephone. The testing was split into four sessions due to the sizeable battery of tasks and questionnaires, and the duration of each session was approximately 45 min. The order of the tasks was randomized within (but not between) the testing sessions, and each session included three to four tasks as well as a few questionnaires. During the course of the computerized phase, three PD participants and nine control participants withdrew from the study. In the PD group, these three participants did not give a reason for their withdrawal (based on the ethical guidelines they did not have to explain why they withdrew). In the control group, three participants mentioned insurmountable time constraints and three participants extra stress, while three participants did not give a reason. The remaining PD group continued the study for a further 6 weeks by participating in an intervention that has been reported earlier (Fellman et al., 2020).

### Working Memory Tasks

WM was assessed with seven computerized tasks tapping on the subdomains selective updating, continuous monitoring, and maintenance of information. In each task, performances of those participants who were extreme outliers (i.e., showing a deviation of at least three times the interquartile range) were removed from the statistical analyses.

#### Selective Updating of Sentences Task (SUS)

In this task (Fellman et al., 2018), Finnish words are presented on the screen in a row of boxes. After 4,000 ms, the words disappear and a blank screen is presented instead. This is followed by an updating stage (lasting 4,000 ms), in which a new row of boxes appears, with some of the boxes containing new words and others being blank. The task is to recall the final sentence formed by the words, considering the updates. The SUS task contained 12 trials (one trial comprised of an initial sentence followed by its updating stages), and the order of trials was randomized for each participant. The 12 trials were split into three blocks, with four trials in each block. The blocks differed in terms of the number of updating stages (two updates, three updates, and five updates) while being similar in terms of sentence length so that one trial of each sentence length (range 4–7 words) was presented in all blocks. The Finnish sentences in the SUS task followed the canonical SVX order, including ordinary declarative sentences of both transitive and intransitive type. The sentences were also designed to be syntactically simple, but the information content concerning the length of the sentence was quite high due to the frequent use of attributes. The stimulus sentences, for instance, included predicative clauses (Koulun loputtua poika oli nälkäinen “After the school ended the boy was hungry”), transitive clauses (Pirteä mies myi koripallon “The cheery man sold a basketball”), intransitive clauses (Nuoret pojat ilahtuivat lahjasta “The young boys were happy for the gift”), ownership clauses (Minun miehelläni on uusi auto “My husband has a new car”), and existential clauses (Perheen talon katolla oli lintu “At the roof of the family’s house there was a bird”). Furthermore, when the sentences were updated with new words, they remained at all times semantically and syntactically plausible. SUS has demonstrated adequate psychometric properties (Fellman et al., 2017).

#### Selective Updating of Digits Task (SUD)

In this task, which was a slightly modified version of the task originally created by Murty et al. (2011), five digits ranging from 0 to 9 are presented on the screen in a row of boxes. After 4,000 ms, the digits disappear and a blank screen (i.e., interstimulus interval) is presented for 100 ms. This is followed by an updating stage (lasting 4,000 ms), in which a new row of boxes appears, with some of the boxes containing new digits and others being blank. As in the SUS task, the participants were thus prompted to replace the old digits with the newly presented digits in the memorized sequence, while maintaining the unchanged digits in memory. This task contained 12 trials, and the order of trials was randomized for each participant. Four of the 12 trials included only the initial sequence without any updates, while four trials also included two updating stages, and yet another four trials five updating stages. The dependent variable was the percentage of correctly recalled digits in the correct order on the updating trials.

#### N-Back Tasks With Digits and Colors (NB-d and NB-c)

The version we developed was based on the classic n-back paradigm (Kirchner, 1958). In this task tapping continuous monitoring of information on the mind, the sequence of stimuli is presented and the task is to respond to whether the currently presented item corresponds to the item presented n items back. The participant responds to each stimulus by pressing on the designated “yes” or “no” button on the keyboard. Two versions of the n-back task were included, one with digits from 1 to 9 and another with colored squares (red, green, blue, yellow, black, and purple). This task contained one-, two-, and three-back conditions, and the order of conditions was randomized for each participant. However, the three-back condition turned out to be too demanding for both groups (i.e., task performance was clearly below chance level). Therefore, we chose to discard the three-back condition from the analyses. All three levels included 48 items of which 16 were targets and 32 non-targets. Half of the non-targets were lures, i.e., stimuli presented just before or after the target. The dependent variable was accuracy as measured by d-prime, and this was calculated separately for the two levels.

#### Forward Simple Span Tasks With Digits and Colors (FSS-d and FSS-c)

This task was based on the classic simple span paradigm (Wechsler, 1997). In the digit task, sequences ranging from 4 to 10 digits in length are presented on the screen. In the color task, we presented colored squares (red, green, blue, yellow, back, and purple) instead of digits. The task is to recall the items in the order they are presented in. The participant responds after each sequence by clicking on the correct items, in a row of horizontally aligned boxes with digits 1–9, on the screen in the correct order. This task contained one trial of each sequence length, and the order of trials was randomized for each participant. The dependent variable was the total number of correctly recalled items in the correct order.

#### Minus 2 Span Task (M2S)

In this task (Salthouse, 1988; Waters and Caplan, 2003), digit sequences of varying length are presented to the participant on the screen. The task is to recall the digits in the order they were presented in, subtract two from each, and return the resulted digits as the response. The participant responds after each sequence by clicking on the final correct digits, in a row of horizontally aligned boxes with digits 1–9, on the screen. This task contained 12 trials, and their order was randomized for each participant. The dependent variable was the total number of correctly recalled items in the correct order. For additional details, see Fellman et al. (2020).

#### Alphabet Working Memory Task (AWM)

In this task (Was et al., 2011), letter sequences of one or two non-adjacent letters are presented to the participant on the screen. Next, instructions for direction (+ or) and number (1, 2, or 3) of transformation are shown. The task is to recall the letter(s) in the order they were presented in, transform them according to the instructions (either add or subtract, either 1, 2, or 3 letters), and return the resulted letter(s) as the response. The participant responds after each sequence by typing the final correct letters in an empty box. This task contained 18 trials, and their order was randomized for each participant. The dependent variable was the proportion of correctly recalled items in the correct order per minute.

#### Running Memory Task (RM)

Based on the paradigm by Pollack et al. (1959), digit sequences of varying length are presented on the screen. The task is to recall the last four digits in the sequence. This task contained eight trials, in a randomized order. The dependent variable used was the total number of correctly recalled items in the correct order.

### Other Computerized Cognitive Tests

#### Simple Reaction Time (SRT)

This task was adapted from the one developed by Mueller (2012). In this task, a square is presented in a fixed spot on the screen, at variable time intervals between 250 and 2,500 ms. The participant’s task is to press the respond key as quickly as possible upon spotting the square stimulus. The dependent variable was the mean reaction time over all responses.

#### Sentence Recall

In this computerized sentence recall task^2^, words of a sentence were presented successively on a screen at a rate of one word per 1,000 ms. Immediately after all the words had been presented, the participant was prompted to reproduce the sentence by typing it in an empty column. The participant was to recall a total of five sentences (length ranging from 18–22 words) that were presented in a randomized order. The proportion of correctly recalled words, regardless of the order they were recalled in, was used as the outcome variable. See Fellman et al. (2017) for details.

#### Wordlist Recall

In this task, 10 words were displayed one at a time for 1,000 ms. The task was to memorize each word in correct serial order, and finally recall them by typing down the words in empty columns. The participant completed altogether three trials, each trial consisting of the same 10 words. However, the order of the words in the list was randomized in each trial. We employed a true recall scoring as the dependent variable, that is, the total number of correctly recalled words in the three trials minus repetitions, perseverations, and additions.

#### Continuous Performance Task (CPT)

Following Conners et al. (2003), we included the CPT in our pre-post battery. In this task, 360 letters appeared on the computer screen, one at a time, for 250 ms. The 360 trials were presented in 18 consecutive blocks of 20 trials. The 18 blocks had different interstimulus intervals (ISIs) of either 1, 2, or 4 s. The ISIs were block-randomized so that all three ISI conditions would occur in every three blocks but in a different order. The participant was to press the spacebar when any letter except the letter “X” appeared on the computer screen. The percentage of trials with letters other than “X” was 90%, and this percentage was constant across all blocks. As the outcome variable, we used the rate of commission errors from the 18 consecutive blocks, that is, the number of times when the participant pressed the spacebar when the letter “X” was presented.

#### Stroop

We employed a computerized Stroop task similar to task versions that have been used in several previous studies (e.g., Salo et al., 2001; Bartsch and Kothe, 2016). In each trial, the participant was shown two stimulus rows on the computer screen: in the upper one, a word (or, in the neutral condition, a series of X) was written in color (red, green, blue, or yellow), and in the lower row there was a word naming the color, written in black. The task was to decide if the font color of the upper row matched the color name in the bottom row. If it matched, the participant had to press the “down” button on the computer keyboard, and if not, to press the “right” button. There were three conditions: neutral, congruent, and incongruent. The participant completed two blocks of each condition in the following order: (1) neutral; (2) congruent; (3) incongruent; (4) incongruent; (5) congruent; and (6) neutral. Each block was comprised of 24 trials, and trial order was randomized within each block. Of the 24 trials, 12 were match trials and 12 no-match trials. The dependent variable was the interference score, calculated as Neutral mean RT minus Incongruent mean RT.

### Self-rating Measures for Everyday Cognitive Abilities

#### Behavior Rating Inventory of Executive Functioning (BRIEF-A)

In this questionnaire (Roth et al., 2005), 75 items regarding executive function in everyday life are presented. The questions fall into nine subscales: inhibit, shift, emotional control, self-monitor, initiate, WM, plan/organize, task monitor, and organization of materials. The questions are rated on a 0–2 point Likert scale (0 = behavior is never observed, 2 = behavior often observed). The dependent variable was the sum score of the nine subscales, and higher scores indicate more difficulty. The cut-off score for significant difficulty was ≥65, as suggested by Roth et al. (2005).

#### Working Memory Questionnaire (WMQ)

This questionnaire (Vallat-Azouvi et al., 2012), includes 30 items regarding WM function in everyday life. The questions fall into three subscales: short-term storage, attention, and executive functioning. The questions are rated on a 0–4 point Likert scale (0 = “no problem at all,” 4 = “very severe problems in everyday life”). The dependent variable was the sum score of the three subscales, and higher scores indicate more difficulty. WMQ has been shown to demonstrate good psychometric properties with healthy persons (Vallat-Azouvi et al., 2012).

### Self-rating Measures for Affective Symptoms

#### Geriatric Depression Scale-30 (GDS-30)

In this questionnaire (Yesavage et al., 1983), 30 items regarding depressive symptoms are presented. The questions are rated with “yes” or “no” responses. The dependent variable was the sum score of the 30 questions, and higher scores indicate more serious depressive symptoms^3^. GDS-30 has been shown to demonstrate good psychometric properties in healthy elderly populations (Yesavage et al., 1983) as well as in PD patients (Ertan et al., 2005).

#### Lille Apathy Rating Scale (LARS)

This interview tool (Sockeel et al., 2006) includes items regarding clinical apathy. The questions fall into nine subscales: everyday productivity, interests, taking the initiative, novelty-seeking, motivation and voluntary actions, emotional responses, concern, social life, and self-awareness. The dependent variable was the sum score of the questions, and higher scores indicate more serious apathy symptoms. The cut-off score for severe apathy was ≥−9, while scores −16 to −10 were interpreted as signifying moderate apathy and scores −21 to −17 as mild apathy, as suggested by Sockeel et al. (2006). LARS has been shown to demonstrate good psychometric properties with PD patients (Sockeel et al., 2006).

In our study, the first part was conducted within the telephone interview protocol. The participant was asked to respond to two open-answer questions, one regarding the subscale of everyday productivity and the other the subscale of interests. The questions are rated on time taken to reply and on the number and variety of activities mentioned. Time taken to reply and the number and variety of activities mentioned are both scored on a five-point 2-(−2) Likert scale. The participant was also asked an additional closed-answer question regarding interests, more specifically the number of times a week s/he practices the first hobby or pastime mentioned. This question is rated on a three-point 1-(−1) Likert scale. The remaining part was conducted as a computerized questionnaire. The participant was asked to respond to 28 questions, four regarding each of the remaining subscales. The questions are rated on a three-point 1-(−1) scale.

### Measures of Global Cognitive Abilities

#### TELE

In this interview protocol (Gatz et al., 1995), 17 items, including altogether 23 questions, screening for early dementia are presented. The items assess orientation to personal information and time, long-term memory, short-term memory, and abstraction. The questions and tasks are rated either on a 0–0.5 point scale (0 = incorrect, 0.5 = correct) or on a 0–1 point scale (0 = incorrect, 1 = correct), depending on the item. The dependent variable was the sum score (maximum: 20 points), and lower scores indicated more difficulty. The cut-off score for dementia was ≤16 as suggested by Gatz et al. (2002), who reported good sensitivity (0.86) and specificity (0.90) for this cut-off. TELE has also been shown to exhibit good sensitivity and specificity for detecting cognitive impairment among aging Finnish subjects and to correlate with clinician-made assessments (Järvenpää et al., 2002).

#### TICS-m

This interview protocol (Welsh et al., 1993) consists of 12 items, including altogether 45 questions, that are used in screening for early dementia. The items tap on cognitive domains affected by dementia, including orientation to personal information and time, long-term memory, receptive and expressive language functions, immediate verbal memory, calculation, and verbal abstraction. The questions and tasks are rated either on a 0–1 point scale (0 = incorrect, 1 = correct) or on a 0–2 point scale (0 = incorrect, 1 = partially correct, 2 = correct), depending on the item. The dependent variable was the sum score (maximum: 50 points), and lower scores indicated more difficulty. The cut-off score for dementia was ≤27, while scores 28–31 were interpreted as signifying MCI, as suggested by Knopman et al. (2010), who reported good sensitivity (0.71 for MCI vs. normal cognition, and 0.69 for dementia vs. MCI) and specificity (0.78 for MCI vs. normal cognition, and 0.71. for dementia vs. MCI) for these cut-offs. TICS-m, too, has also been shown to exhibit good sensitivity and specificity among aging Finnish subjects and to correlate with clinician-made assessments (Järvenpää et al., 2002).

### PD-Related Scales and Measures

#### UPDRS Parts I and II

In the UPDRS rating scale (Fahn and Elton, 1987), items relevant to the clinical status of PD patients are presented. The scale is divided into six subscales. The assessment of the first two subscales is based primarily on the information given by the patient or their family, while the other four subscales are assessed by a physician. Due to the fully home-based nature of the present study, only the subscales I and II were administered in the telephone interview (part III requires an observational assessment of motor functions). Part I includes four questions about mentation, behavior, and mood. Part II includes 13 questions about activities of daily living. The items are rated on a 0–4 point Likert scale (0 = normal or no symptoms, 4 = severe symptoms). The dependent variables were the sum scores for both scales separately.

#### Parkinson’s Disease Questionnaire-39 (PDQ-39)

In this questionnaire (Jenkinson et al., 1997), 39 items representing PD-specific health status are presented. The items assess eight dimensions of functioning and well-being: mobility, activities of daily living, emotions, stigma, social, cognition, communication, and body pain. The questions are rated on a 0–4 point Likert scale (0 = difficulty is never experienced, 4 = difficulty is always experienced or unable to complete the action in question). The dependent variable was the sum score of the eight dimension scores, and higher scores indicate more difficulty. PDQ-39 has been shown to exhibit high internal consistency and test-retest reliability with PD patients living at home (Peto et al., 1997). It has also been found to concur with clinician-made assessments as well as general health quality measures.

#### Self-assessment Parkinson’s Disease Disability Scale (SPDDS)

This questionnaire (Brown et al., 1989) consists of 25 items that represent PD-specific disability status. The items tap on two dimensions of activities of daily living: gross mobility and fine co-ordination. The questions are rated on a 1–5 point Likert scale (1 = independently and without difficulty, 4 = unable to complete the action in question). The dependent variable was the sum score, and higher scores indicate more difficulty. SPDDS has been shown to have high internal consistency with PD patients living at home (Biemans et al., 2001). It has also been found to concur with clinician-made assessments as well as general disability measures.

#### Other Clinical Measures

A total daily levodopa equivalent dose (LED) was calculated for each PD participant using the formulae provided by Tomlinson et al. (2010). LED represents the summary of antiparkinsonian drugs the patient receives and takes into account the intensity of different mediations (Tomlinson et al., 2010). As levodopa improves symptomatic control but also causes some complications, reporting LED values is recommended. All except one PD participant used levodopa during the cognitive assessment (LED mg/day range: 0–1,364).

### Measures of Motivation and Alertness

The level of motivation and alertness were asked for at the end of each testing session, each with a single question (“How motivated were you while completing the tasks?” (translated from the Finnish item); “How alert are you feeling at the moment (translated from the Finnish item).” The level of motivation and alertness were each rated on a 1–5 Likert scale (1 = “not at all motivated” or “very tired,” 5 = “very motivated” or “very alert”). The dependent variables were the mean scores of the four sessions, and higher scores indicate higher motivation or alertness.

#### Analytical Approach

##### Background Characteristics and Group Comparisons for Single Measures

The two groups (PD vs. control) were compared on background characteristics. The data was at first screened for normality with Shapiro–Wilkinson’s test at the significance level of 0.05. Independent *t*-tests were then used for analyses for normally distributed continuous variables: *U*-tests for ordinal categorical variables and non-normally distributed continuous variables and Chi-squared tests for nominal categorical variables. This was followed by an examination of possible group differences (PD vs. controls) in the cognitive tasks and self-reported measures. This was established by conducting independent-samples *t*-tests for every single measure independently. For effect sizes, Cohen’s *d* values with group means (M) and pooled standard deviation (SDp) were calculated, using the following formula: *d* = (M_PD_ − M1_Controls_)/SDp, in which ${\text{SD}}_{\text{p}}=\surd \left[\left(\left({\text{n}}_{1}-1\right){\text{s}}_{1}^{2}+\left({\text{n}}_{2}-1\right){\text{s}}_{2}^{2}\right)/\left({\text{n}}_{1}+{\text{n}}_{2}-2\right)\right]$.

##### Between-Group Analysis

For examining possible group differences in WM functioning, self-rated cognition, and self-rated mood in the PD vs. the control group on the latent level, we employed confirmatory factor analyses (CFA) using multiple indicators multiple causes (MIMIC) modeling, also referred to as SEM. A MIMIC model is otherwise similar to the CFA, except that the former one comprises a dichotomous observed variable (e.g., group membership) on which the latent variables can be regressed on (e.g. Kline, 2011). Given our sample size, the MIMIC model was deemed as most feasible for the present study (see e.g., Breitsohl, 2019). Furthermore, we minimized the number of parameters and excluded extreme outliers (values three times the interquartile range above or below the first or the third quartile) to achieve normally distributed data (see Supplementary Table S3 for information on the original data distribution, and Supplementary Table S4 for information on the updated data distribution after outlier exclusion). Both of these operations were expected to help in obtaining robust models (see SOM for details of the models).

**Figure 2:**
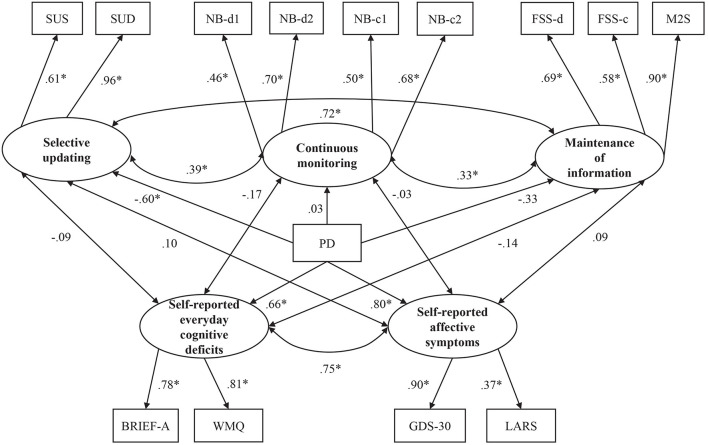
Standardized coefficients for the working memory (WM) model. Latent constructs are shown in ellipses, and observed variables are shown in rectangles. SUS, Selective Updating of Sentences task; SUD, Selective Updating of Digits Task; NB-d1, N-Back task with digits level 1; NB-d2, N-Back task with digits level 2; NB-c1, N-Back task with colors level 1; NB-c2, N-Back task with colors level 2; FSS-d, Forward Simple Span task with digits; FSS-c, Forward Simple Span task with colors; M2S, Minus 2 Span task. *p < 0.05.

**Figure 3:**
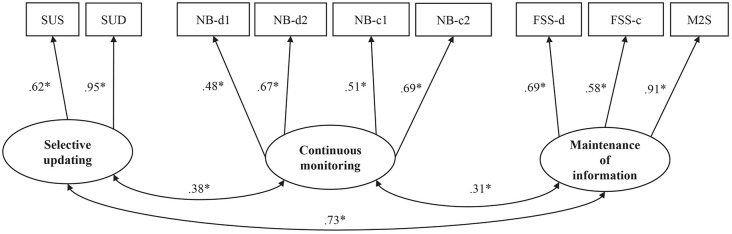
Standardized coefficients for the model of WM, affective symptoms, and everyday cognitive deficits. Latent constructs are shown in ellipses, and observed variables are shown in rectangles. PD, group covariate (1 = PD, 0 = control group); SUS, Selective Updating of Sentences task; SUD, Selective Updating of Digits task; NB-d1, N-back task with digits level 1; NB-d2, N-Back task with digits level 2; NB-c1, N-Back task with colors level 1; NB-c2, N-Back task with colors level 2; FSS-d, Forward Simple Span task with digits; FSS-c, Forward Simple Span task with colors; M2S, Minus 2 Span task; BRIEF-A, Behavior Inventory of Executive Functioning; WMQ, Working Memory Questionnaire; GDS-30, Geriatric Depression Scale-30; LARS, Lille Apathy Rating Scale. *p < 0.05.

Following our CFA assessment of WM (see Figure 2 and Supplementary Table S1), we added two latent variables to the model: self-report measures (see Figure 3) and the dichotomous group covariate consisting of the PD group (coded as 0) and the control group (coded as 1). The latent variables for self-report measures were constructed according to theoretical assumptions and available data, with an *everyday cognition* factor consisting of BRIEF and WMQ, and an *affective symptoms* factor consisting of GDS-30 and LARS. Lastly, paths were drawn from each latent factor (i.e., the three subdomains of WM and the two domains of self-report measures) to the group covariate.

For model estimation with the MIMIC analyses, we used the maximum likelihood robust (MLR) technique to adjust for non-normality (Muthén and Muthén, 1998–2012) due to a slight skewness in some of our variables and our relatively modest sample size. For model fit evaluation (see SOM for details), we used the Chi-square and multiple fit indices: the standardized root mean square residual (SRMR), the root-mean-square error approximation (RMSEA), Bentler’s comparative fit index (CFI), and the Tucker-Lewis index (TLI). The Chi-square assesses the magnitude of discrepancy between the sample and fitted covariance matrices (Hu and Bentler, 1999). The approximate cut-off values for a relatively good fit are >0.95 for both CFI and TLI. In turn, SRMR and RMSEA are absolute fit indices, measuring how well a model reproduces the sample data. In other words, the fit of the proposed model is the degree of departure from the perfect fit of zero. The approximate cut-off values for a relatively good fit are <0.08 for SRMR and <0.06 for RMSEA. In the MIMIC model, the standardized regression coefficients (β) serve as effect sizes, as these values can be directly interpreted as correlation coefficients in the models (Muthén and Muthén, 1998–2012). All CFA and SEM analyses were run in Mplus version 7.2.

#### Within-Group Analyses

To further examine the role of WM in different aspects of the PD symptomatology, we also examined whether the PD patients’ cognitive status and disease severity possessed some predictive power in any of the three subdomains of WM. As our PD group (*n* = 52) was underpowered for a within-group SEM analysis, we examined these relationships at a non-latent level. For WM functioning, we created composite scores for each subdomain of WM so that the *selective updating* subdomain comprised of the averaged *z-transformed* scores of the SUS and SUD, whereas the *continuous monitoring* subdomain included the averaged *z-scores* of one- and two-back tasks with digits and colors. The *maintenance* subdomain, in turn, entailed the averaged *z-scores* of the Forward simple span tasks with digits and colors, and the Minus 2 span task. Thus, these three outcome variables served as composite scores of the three subdomains of WM identified in the CFAs. As regards the self-report measures, we created two composite scores. One of these composite scores, titled *global cognition*, consisted of the scores from TICS-M and TELE which were standardized and averaged together. The other composite, titled *disease severity*, was based on UPDRS 1, UPDRS 2, PDQ-39, and SPDDS scores using an identical calculation as described above. These composite scores for the self-reported measures were deemed adequate considering the statistically significant intercorrelations between the measures included in the global cognition (*r* = 0.37) and disease severity (*r* = 0.55–0.74; see also Supplementary Table S5). Due to the modest sample size for within-group analyses, the associations between WM performance and the self-report predictors were assessed using simple linear regression analyses. Given that the three WM composite scores served as dependent variables, we ran three separate analyses for each predictor of interest. The regression analyses were performed in the R environment (version 3.5.2, R Core Team, 2013).

## Results

### Background Characteristics and Group Comparisons for Single Measures

The groups were comparable on all demographic characteristics, as well as on motivation and alertness evaluations throughout the test sessions (Table 1). The age of the PD group ranged from 45 to 72 years (*M* = 65.1, SD = 5.5) and they had an average education of 14.8 years (SD = 4.8). The mean age at disease onset had been 59.5 years (SD = 7.2), while the average disease duration had been 5.6 years (SD = 4.8). The age of the control group ranged from 50 to 73 years (mean 66.0, SD = 4.1) and they had an average education of 14.2 years (SD = 3.8).

There were some differences between the groups in cognitive performance, global cognitive abilities, self-reported everyday cognition, and self-reported affective symptoms (Table 1). As shown in Table 2, the PD patients performed significantly worse than the healthy controls on SRT (*t* = 4.70, *p* < 0.01, *d* = 0.93), CPT (omission errors, *t* = 3.26, *p* < 0.01, *d* = −0.68; commission errors, *t* = 2.85, *p* = *0*.01; *d* = −0.56), and the Wordlist recall task (*t* = 2.18, *p* < 0.05; *d* = 0.42). Sentence recall was the only non-WM task that systematically correlated with the WM tasks within the PD group (see Supplementary Tables S6–S8).

**Table 2:** Group differences for computerized tasks between the PD patients and the healthy controls.

		PD patients		Healthy controls					
Measure	Dependent variable	*M*	*SD*	*M*	*SD*	*df*	*t*	*p*	Cohen’s *d*
SUS	Percentage of correctly recalled items	57.93	17.67	62.61	19.52	103	−1.29	0.20	0.25
SUD	Percentage of correctly recalled items	63.97	25.76	77.77	17.07	103	−3.23	<0.01	0.63
NB-d1	d-prime score	2.35	0.90	2.59	1.14	101	−1.17	0.25	0.23
NB-d2	d-prime score	1.28	0.64	1.18	0.72	101	0.69	0.50	0.15
NB-c1	d-prime score	2.66	0.88	2.69	0.99	102	−0.19	0.85	0.03
NB-c2	d-prime score	1.33	0.70	1.34	0.79	102	−0.08	0.94	0.01
FSS-d	Sum of correctly recalled items	24.27	7.03	26.19	7.66	104	−1.34	0.18	0.26
FSS-c	Sum of correctly recalled items	22.23	5.14	23.02	7.42	104	−0.64	0.53	0.12
M2S	Sum of correctly recalled items	40.87	13.30	44.39	9.96	104	−1.55	0.13	0.30
AWM	Accuracy/latency	1.66	0.86	1.80	0.85	104	−0.85	0.40	0.16
RM	Sum of correctly recalled items	19.69	7.21	18.91	8.51	104	0.51	0.61	0.10
SRT	Mean reaction time	396.23	41.85	358.04	40.54	101	4.70	<0.01	-0.93
CPT	Sum of commission errors	12.37	7.57	8.85	4.68	103	2.85	0.01	-0.56
CPT	Sum of omission errors	2.26	3.64	0.48	0.83	93	3.26	<0.01	-0.68
Stroop	Interference score	245.03	246.28	201.81	218.56	96	0.92	0.36	-0.19
Sentence recall	Percentage of correctly recalled items	59.17	14.09	63.53	13.25	103	−1.63	0.11	0.32
Word list recall	Sum of correctly recalled items	18.38	4.33	20.09	3.74	104	−2.18	0.03	0.42

*Note. SUS, Selective Updating of Sentences task; SUD, Selective Updating of Digits task; NB-d1, N-Back task with digits level 1; NB-d2, N-Back task with digits level 2; NB-c1, N-Back task with colors level 1; NB-c2, N-Back task with colors level 2; FSS-d, Forward Simple Span task with digits; FSS-c, Forward Simple Span task with colors; M2S, Minus 2 Span task; AWM, Alphabet Working Memory task; RM, Running Memory task; SRT, Simple Reaction Time task; CPT, Continuous Performance Task*.

The PD patients exhibited also general cognitive impairment (TICS-m, *p* < 0.01, *d* = 0.68), and reported more everyday cognitive difficulties (WMQ, *p* < 0.01, *d* = −0.61; BRIEF-A, *p* = 0.02, *d* = −0.45) as well as depressive symptoms (GDS-30, *p* < 0.01, *d* = −0.69) as compared to the controls. The PD patients did not, however, differ from the controls on the other global cognitive ability measure (TELE, *p* = 0.67, *d* = 0) or on the self-reported apathy rating (LARS, *p* = 0.48, *d* = −0.22).

Although no participants showed cognitive impairment reaching the dementia cut-off scores, according to the TICS-m criteria (see “Material and Methods” section) two PD patients and one healthy control scored for MCI. Eight PD patients and two controls also had mild depression symptoms (GDS scores 11–20), but moderate or severe symptoms (GDS scores ≥21) were not found in either group. One PD patient and two healthy controls scored for mild apathy while moderate apathy was found in one PD participant. Seven PD participants and four controls expressed significant subjective difficulties of executive functioning.

### SEM Analyses on the WM Partition Into Three Subdomains

Using CFA, we first attempted to form a factor model of WM functioning that exhibited the best-fit indices. We first formed three latent factors of WM functioning titled *continuous monitoring*, *selective updating*, and *maintenance of information*. We examined altogether 26 alternative WM factor models where we let our 11 WM tasks to load together in different constellations under either of the three latent factors (see Supplementary Table S1) see also Figure 2). Out of the 26 alternative CFA models depicting WM, model 12 had the best fit (see Supplementary Table S2), fulfilling the standards for a good fit by almost all goodness-of-fit indices, with only TLI (0.941) being slightly below the recommended minimum value (0.95). Thus, this model was chosen for further analyses. The model adds the SUS and SUD to the selective updating factor, the *n-back* tasks (digits, colors) to the continuous monitoring factor, and the forward span tasks (digits, colors) and the M2S task to the maintenance factor (see Figure 2 for the standardized coefficients, see also Supplementary Table S9 for the loadings of items on their respective latent factors and Supplementary Table S10 for the standard errors and confidence intervals of the parameter estimates). For the rest of the WM tasks (i.e., RM, and AWM) that were excluded from the final model and those cognitive tasks not taxing WM, we report independent-samples *t*-tests in which the outcome variable of interest served as the dependent variable and group (PD vs. controls) as the independent variable (see Table 2 in the “Results” section).

### Working Memory, Affective Symptoms, and Everyday Cognition

The MIMIC model with the group as a covariate tested the effect of PD on task-based WM, self-rated affective symptoms, and self-rated everyday cognitive difficulties, as well as the connections between these variables (see Figure 3 for the standardized coefficients, see also Supplementary Tables S11, S12 for zero-order correlations between the indicator variables and Supplementary Table S13 for the standard errors and confidence intervals of the parameter estimates). The model fit was good, Chi-Square (*p* = 0.0896; *χ*^2^/*df* = 1.239), and RMSEA (0.047), CFI (0.959), as well as SRMR (0.058) were all within the desired range. Only TLI (0.940) was slightly below the recommended value (0.95).

Group (PD vs. control) had a significant effect on task performance in one of the WM domains, namely selective updating (*p* < 0.01, *β* = −0.60). Having PD predicted lower performance on tasks of selective updating. PD did not, however, predict performance on tasks tapping continuous monitoring (*p* = 0.91, *β* = 0.03) or maintenance of information (*p* = 0.10, *β* = −0.33), although there was a trend towards statistical significance for maintenance. The performance on tasks of the three WM subdomains was significantly correlated (selective updating and continuous monitoring, *p* = 0.01, *β* = 0.39; maintenance of information and continuous monitoring, *p* = 0.01, *β* = 0.33; maintenance of information and selective updating, *p* < 0.01, *β* = 0.72). Additionally, there was a significant group effect on self-rated affective symptoms (*p* < 0.01, *β* = 0.80) as well as on self-rated cognitive dysfunction (*p* < 0.01, *β* = 0.66). Hence, the PD patients reported more affective symptoms and cognitive dysfunction than the controls. The amount of self-rated affective symptoms was also correlated with the amount of self-rated cognitive dysfunction (*p* < 0.01, *β* = 0.75). Neither the amount of self-rated affective symptoms nor self-rated cognitive dysfunction correlated with any WM subdomain.

### WM as a Function of Global Cognition and Disease Severity Within the Patient Group

**Figure 4:**
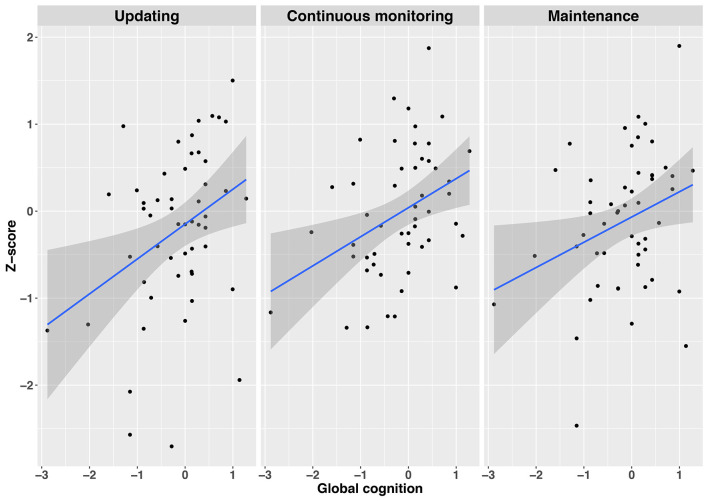
Regression plots depicting the relationship between global cognition and the three WM subdomains Updating (left panel), Continuous monitoring (mid panel), and Maintenance (right panel). Gray shaded regions represent 95% confidence intervals on the slope. Note. All variables are *z*-standardized.

Employing simple regression analyses, we examined whether global cognition and disease severity had some predictive value on the three subdomains of WM functioning (see also Supplementary Tables S14, S15 for intercorrelations). Global cognition consisted of a standardized composite score of TELE-M and TICS, whereas disease severity consisted of a standardized composite score of UPDRS I and II, PDQ-39, and SPDDS. The three WM subdomains included the WM tasks with the best fit indices as identified in the CFAs (selective updating, continuous monitoring, and maintenance of information), being standardized and averaged within their respective subdomain. Global cognitive abilities predicted performance in selective updating (*R*^2^ = 0.12, *β* = 0.35, *t*_(52)_ = 7.07, *p* = 0.011), continuous monitoring (*R*^2^ = 0.14, *β* = 0.37, *t*_(52)_ = 8.09, *p* = 0.006), and maintenance (*R*^2^ = 0.07, *β* = 0.30, *t*_(51)_ = 4.99, *p* = 0.030) domains, indicating that those with a better global cognitive status performed better in all subdomains of WM, as compared to those with a poorer global cognitive status (see also Figure 4). Disease severity did not predict performance either in the continuous monitoring (*R*^2^ = −0.01, *β* = 0.08, *t*_(52)_ = 0.29, *p* = 0.59), maintenance of information (*R*^2^ = 0.03, *β* = −0.17, *t*_(52)_ = 1.47, *p* = 0.23) or in selective updating (*R*^2^ = 0.05, *β* = −0.23, *t*_(52)_ = 2.65, *p* = 0.11) domains.

## Discussion

This study was set out to examine which subdomains of WM are particularly affected in PD, and to discover how WM is associated with various other factors, including everyday cognitive deficits, other executive functions, psychiatric and PD-related symptoms, and early cognitive impairments. As our online assessment required relatively well-preserved motor skills, only PD patients with mild to moderate symptoms volunteered for participation. Nevertheless, our participants showed typical PD characteristics, such as difficulties in several cognitive tasks, subclinical depression symptoms, as well as global cognitive impairment. Participants were well-motivated (mean 4.1/5) and also relatively alert (mean 3.6/5), and task performances appeared to be quite stable and comparable to prior laboratory-based studies in aged participants (see Fellman et al., 2020). We were also able to replicate several well-studied effects, such as the *n*-back load effect, Stroop effect, omission/commission effects (CPT), and WM span effect, providing further evidence of the data quality.

### Working Memory Deficits in Parkinson’s Disease

The model that included latent factors reflecting three WM subdomains (updating, continuous monitoring, and maintenance of information) provided a good fit to the data. As for the group differences in the WM tasks, PD was only predictive of selective updating (Figure 2). For the maintenance of information, there was a trend pointing for a possible weak group difference. Continuous monitoring of WM, in turn, did not show a group difference. Our findings that WM deficits in PD are most clearly observed in the subdomain of updating support our hypothesis that was based on the prominent role of the striatum in PD (Owen et al., 1998), as well as its carefully examined contribution to WM updating (Frank et al., 2001; O’Reilly and Frank, 2006). Due to the role of WM updating in n-back tasks, we expected to see group-level differences in this domain as well, but this was not the case. However, a closer examination of the intercorrelations indicates that selective updating tasks and n-back tasks taps on different WM domains also in terms of their association with non-WM measures (see below). One possible explanation for the lack of correlation between the two updating-related tasks could be that the n-back tasks can be performed using strategies that do not necessarily require updating (e.g., intermittent chunking) while performing the selective updating tasks accurately is not possible without updating the WM contents.

As expected (second hypothesis), we observed a correlation between subclinical cognitive impairment and WM performance also within the PD group. Consistent with the shared role of selective updating and continuous monitoring tasks, these two domains were both associated with global cognitive abilities. However, also the performance in the maintenance task was associated with the global cognitive status. Together these findings suggest that PD hampers limited aspects of WM, but more widespread problems are observed in case PD is coupled with a global decrease in cognitive status. The relatively limited WM deficits directly associated with mild-to-moderate PD is also supported by a weak link between WM performance and PD symptoms. Despite difficulties in some non-WM cognitive tasks, only selective updating tasks showed a weak trend pointing to a possible direct link to PD symptoms (Figure 4). Finally, the measures of processing speed (SRT), attention and motor inhibition (CPT), as well as wordlist memory recall in which PD patients also performed worse than healthy controls (Table 1), were not correlated with WM performance (see Supplementary Table S7), further implying that WM represents a separate cognitive domain affected in PD.

Taken together, in this particular population of PD patients with mild-to-moderate symptoms WM difficulties were not associated with other problems, unless global cognitive abilities were affected. Nevertheless, the present study provides important information on the WM subdomain function in PD, mainly manifesting in impaired updating. To our knowledge, this has not been addressed in prior PD studies, even though theoretical studies have implicated updating as the key WM deficit in striatal dysfunction (Frank et al., 2001). Future research should test whether WM updating could serve as a valuable early-onset cognitive marker in PD, whereas other WM subdomains could be more useful only in later stages of the disease or when more global cognitive impairment takes place.

### Associations Between Cognitive Deficits and Psychiatric Symptoms

Neither everyday cognition nor psychiatric symptoms were correlated with WM performance in the PD group. These findings support the existence of subjective cognitive decline in PD (see Erro et al., 2014; Aarsland et al., 2017), suggesting that objectively measured cognitive performance, subjectively experienced cognitive problems, and affective symptoms arise from distinct underlying factors. This is the case in WM as well. Lack of correlation between subjective cognitive complaints and task performance has also been observed in some previous studies with other cognitive measures (Marino et al., 2009; Dujardin et al., 2010; Copeland et al., 2016). Our study further indicates that both everyday cognitive difficulties and affective symptoms are linked to PD symptoms, indicating that the various types of difficulties in daily living could have a common origin that perhaps relates to psychiatric well-being. Cognitive difficulties directly related to PD and those associated with a decline in global cognitive abilities could, in turn, have different underlying mechanisms. As noted above, our findings suggest a direct link between PD and WM updating, while more widespread WM deficits may occur in parallel with global cognitive decline.

In keeping with some previous studies (Marino et al., 2009; Lehrner et al., 2014; Santangelo et al., 2014; Hong et al., 2018), we observed a correlation between affective symptoms and everyday cognitive deficits. Such findings have also been reported in other dementing disorders (Hendrie et al., 1997) as well as in healthy aging (Balash et al., 2013). As suggested in several previous studies (Marino et al., 2009; Sitek et al., 2011; Santangelo et al., 2014; Castro et al., 2016), affective symptoms could worsen the subjective perception of cognitive deficits in PD patients. Another possibility is that facing cognitive difficulties in everyday situations gives rise to depressive symptoms or apathy, but such cognitive problems were not captured by the task battery used in the present study. Regardless of the direction of effect, both subjective cognitive complaints and affective symptoms could be precursors for cognitive impairment that should be taken seriously and monitored (Hong et al., 2014; Lehrner et al., 2014). To conclude, PD patients typically have difficulties in several objectively measured cognitive tasks and they also report difficulties in their everyday lives. However, although they may be aware of their global cognitive impairments, they do not recognize their actual WM deficits.

### Clinical Value of the Present Findings

Our PD participants had problems in WM updating that were unrelated to their other cognitive difficulties, such as prolonged response latencies, difficulties in maintaining attention and inhibiting irrelevant information, and episodic memory deficits. As compared to other WM subdomains, updating was the only subdomain that clearly discriminated PD patients from healthy controls even when no other problems were considered. Altogether, these findings suggest that the WM subdomain of updating should receive particular attention when surveying onset cognitive deficits in PD patients. However, further test development is needed to utilize this knowledge in practice, as standardized, readily available measures of WM updating are lacking.

The importance of regularly monitoring cognitive impairments with objective measures is further supported by the patients’ lack of self-awareness for their actual cognitive performance. So far, it has been challenging to follow up cognitive abilities, since the assessment has relied on regular visits to the clinic. Our study demonstrates that mild-to-moderate PD patients can self-administer computerized cognitive tests at home, which is more cost-efficient and user-friendly than the existing testing practices. Related projects have recently been launched in other dementing illnesses, but the evidence of the feasibility of such methods in PD is scarce (Mackin et al., 2018). The task performance scores obtained from PD patients were as robust as in the healthy controls and aligns well with prior studies (e.g., Waris et al., 2017). Already at the early stages of the disease, PD considerably increases the risk of various cognitive deficits. At this stage when the patients can still live relatively independently, these problems can be assessed with online methods.

### Limitations

It is important to bear in mind that our PD sample does not represent the patient population as a whole. As the study was mainly advertised and conducted online, our participants may have been more experienced and motivated computer and internet users than other PD patients. Our results are also not applicable to PD patients with dementia, severe psychiatric illnesses, neurological illnesses, or other severe health issues. Additionally, the motor skills essential for the present study (use of the computer keyboard and mouse) may be impaired especially in advanced PD. It is possible that some of the effects, such as the lack of associations between task-based cognitive measures and depressive symptoms, could be due to mildness of the symptoms (Santangelo et al., 2014).

Due to the home-based setup, the assessment of PD symptom severity could not rely on observations but was instead based on patients’ responses to our telephone interview questions and online questionnaires. As a result, the severity assessment reflected the patient perspective regarding their PD-related disability, rather than an assessment performed by an expert. It should be noted, however, that the disease severity assessments used in the present study have been shown to correlate strongly with the golden standard UPDRS method (Biemans et al., 2001; Morley et al., 2015), and that 2/3 of the UPDRS interview was conducted. Future online studies could, however, be improved in this respect by administering video-based evaluations of the motor problems. In this study, we selected phone-based interviews, as those are already widely used in dementia research (especially TELE and TICS-m). Video-based methods can be utilized after the protocols have been validated and standardized, and it has been demonstrated that neurologists can conduct reliable assessments with such methods. Besides video-based evaluations, the quality of the functional and clinical assessment could have been improved by including an additional assessment given by a caregiver. As the caregivers can have a highly varying role in the life of the participants (some may not even have a caregiver), and the participants were still relatively well-functioning having only mild-to-moderate symptoms, we nevertheless chose to restrict to self-assessments.

Finally, although our task selection was based on neuroscientific findings on the role of WM updating in frontostriatal functions, we were not able to include MRI or CT images to verify the level of neuronal degeneration in these areas. Such an analysis linking the behavioral data to abnormal brain structure and/or function would allow for stronger conclusions and would be important to examine in future studies.

## Conclusion

Our study sheds light on the nature of WM deficits in PD and elaborates on the interplay of WM, subjective experiences of cognitive difficulty, and self-rated affective symptoms. The results indicate that WM deficits in PD involve mainly the subdomain of updating, possibly resulting from the underlying dysregulation of the frontostriatal networks. Additionally, our results point to strong connections between subjective cognitive deficits and affective symptoms in PD, even when large-scale performance-based WM deficits are not present. Neither self-ratings of affective symptoms nor cognitive defects were associated with WM task performance, suggesting that objectively measured performance and subjective evaluation relate to different phenomena. Besides the links between task-based WM updating and global cognitive impairments, we observed weak associations between cognitive functioning and overall disease severity, suggesting that cognitive impairment in mild-to-moderate PD might are relatively well-preserved. To our knowledge, this is the first study to use SEM for examining the links between PD, self-rated cognitive deficits, self-rated affective symptoms, and WM subdomains. Finally, we demonstrated that computerized online assessment is a potential low-cost and easily administered tool that can be used with early-stage PD patients. In the future, similar online platforms could be utilized in monitoring the evolvement of WM deficits in PD across time.

## Data Availability Statement

The raw data supporting the conclusions of this article will be made available by the authors, without undue reservation.

## Ethics Statement

The studies involving human participants were reviewed and approved by Ethics Committee of the University of the Hospital District of Southwest Finland. The patients/participants provided their written informed consent to participate in this study.

## Author Contributions

JS, ML, JR, UE, and DF designed the experiment. DF and LR recruited the patients and collected the data. JR went through the clinical interviews when necessary. LR and DF performed the analyses under the supervision of JS. ML, UE, and JR commented on the data-analyses. JS and LR wrote the manuscript that was commented, complemented, or agreed on by all authors.

## Conflict of Interest

JR serves as a consultant for CRST Ltd (Clinical Research Services Turku). The remaining authors declare that the research was conducted in the absence of any commercial or financial relationships that could be construed as a potential conflict of interest.

## Footnotes

^1^www.vtkl.fi/seniorsurf

^2^As the present Sentence Recall task is a supraspan task (18–22 to-be-remembered words per sentence), we did not include it as a WM maintenance task in the SEM analyses. Albeit sentence processing clearly engages WM, there is also a considerable linguistic processing component involved (for older adults and children, see Jefferies et al., 2004 and Klem et al., 2015; respectively).

^3^https://sumut.fi/wp-content/uploads/2020/02/Geriatrinen-depressioasteikko-GDS.pdf
