# Supplementary material for: Disentangling the Role of Working Memory in Parkinson’s Disease
Source: Front Aging Neurosci. 2020 Sep 25;12:572037. doi: 10.3389/fnagi.2020.572037 (PMC7544957; doi:10.3389/fnagi.2020.572037)
Supplement: Supplementary file 1 [file Data_Sheet_1.doc]

**Article title**

Disentangling the role of working memory in Parkinson’s disease

**Authors' names, degrees and affiliations**

Dr. Juha Salmi ^1,2,3,4*^, M.A. Liisa Ritakallio ^1,3*^, Dr. Daniel Fellman ^3,5*^, Dr. Ulla Ellfolk ^3,6^, Prof. Juha O. Rinne ^7,8^, Prof. Matti Laine ^3,9^

^1^Department of Psychology and Speech-Language Pathology, University of Turku, Turku, Finland

^2^Turku Institute for Advanced Studies, University of Turku, Finland

^3^Department of Psychology, Åbo Akademi University, Turku, Finland
^4^Department of Psychology and Logopedics, University of Helsinki, Helsinki, Finland

^5^Department of Psychology, Department of Psychology, Umeå University, Umeå, Sweden

^6^Department of Psychiatry, Visby County Hospital, Visby, Sweden
^7^Division of Clinical Neurosciences, Turku University Hospital, Turku, Finland
^8^Turku PET Centre, University of Turku, Finland
^9^Turku Brain and Mind Center, University of Turku, Finland

*authors contributed equally

**Corresponding author**
Dr. Juha Salmi

Department of Psychology and Logopedics
FI-20014, University of Turku

FINLAND

Tel: +358 40 511 8678, E-mail: juha.salmitaival@utu.fi

## Supplementary Online Materials

## Participants

The participants were offered no financial compensation for their participation. However, they were informed about the option to ask for feedback on their performance in the study.

## Design and procedure

The telephone interview began with questions related to the practical aspects of participation, such as whether the participant had computer and Internet access at home and whether they felt they had an adequate amount of computer experience and enough time to be able to complete the computer testing at home. The participants were instructed to complete the online testing sessions on different weekdays to avoid tiredness. The participants were, however, free to choose the days and the times for the sessions.

## Statistical analyses

**Specification of model and evaluation of fit.** For model estimation with all CFA and MIMIC analyses, we used the maximum likelihood robust (MLR) technique. This refers to the way the specified latent variable loadings are estimated, based on the covariance matrix. MLR estimation was chosen, as it adjusts for non-normality (Muthén & Muthén, 2012). This was deemed the best option for our data, as our sample size is on the smaller side from a SEM perspective and as some variables were bordering on the skewness and kurtosis limits set for SEM. For model fit evaluation, we used the Chi square and multiple fit indices: the standardized root mean-squared residual (SRMR), the root mean-square error approximation (RMSEA), Bentler’s comparative fit index (CFI), and the Tucker-Lewis index (TLI). The Chi square assesses the magnitude of discrepancy between the sample and fitted covariance matrices (Hu & Bentler, 1999). The approximate cut-off values for a relatively good fit are more than .95 for both CFI and TLI. SRMR and RMSEA, on the other hand, are absolute fit indices, which measure how well a model reproduces the sample data. In other words, the fit of the proposed model is degree of departure from the perfect fit of 0. The approximate cut-off values for a relatively good fit are less than .08 for SRMR and less than .06 for RMSEA.

**Analysis procedure.** The data was screened for extreme outliers, and values three times the interquartile range, further above the 3^rd^ quartile, or below the 1^st^ quartile were deleted. All 26 alternative WM factor models included three factors: continuous monitoring, updating, and maintenance (see Supplementary Table 1 for structure of the models), but the consistency of the factors varied between the models. The continuous monitoring factor was fixed so that it always consisted of the four n-back variables (NB-d 1-back and 2-back, NB-c 1-back and 2-back). The updating factor always included at least the two selective updating tasks (SUS, SUD). The maintenance factor always included at least the two simple span tasks (FSS-d, FSS-c). However, as we speculated that the three remaining tasks (M2S, AWM, and RM) set demand for both updating and maintenance, we were unsure as to which of these two categories they would better fit, if either. Therefore, we created alternative models for all the possible combinations. This means that all of these tests (M2S, AWM, and RM) could be added into one of these two open factors (updating or maintenance), or that one or two tests could be added into one factor and zero to two tests into the other factor.

**References**

Hu, L.T., & Bentler, P.M. (1999). Cutoff Criteria for Fit Indexes in Covariance Structure Analysis: Conventional Criteria versus New Alternatives. *Structural Equation Modeling*, 6, 1-55. 
 doi.10.1080/10705519909540118

Muthén, L.K., & Muthén, B.O. (1998-2012). Mplus User’s Guide (Seventh Edition). Los Angeles, CA: Muthén & Muthén. (Released 2012)
