# Supplementary material for: Disentangling the Role of Working Memory in Parkinson’s Disease
Source: Front Aging Neurosci. 2020 Sep 25;12:572037. doi: 10.3389/fnagi.2020.572037 (PMC7544957; doi:10.3389/fnagi.2020.572037)
Supplement: Supplementary file 2 [file Data_Sheet_2.docx]

**Supplementary Tables**

| **Supplementary Table 1**. Proposed Factor Structures for the Working Memory Model (*N* = 106). | | | |
| --- | --- | --- | --- |
|  |  |  |  |
| Model | Factor structure | | |
|  | Selective updating | Continuous monitoring | Maintenance of information |
| 1 | SUS, SUD, M2S, AWM, RM | NB-d1, NB-d2, NB-c1, NB-c2 | FSS-d, FSS-c |
| 2 | SUS, SUD, M2S, AWM | NB-d1, NB-d2, NB-c1, NB-c2 | FSS-d, FSS-c |
| 3 | SUS, SUD, RM, AWM | NB-d1, NB-d2, NB-c1, NB-c2 | FSS-d, FSS-c |
| 4 | SUS, SUD, RM, M2S | NB-d1, NB-d2, NB-c1, NB-c2 | FSS-d, FSS-c |
| 5 | SUS, SUD, M2S | NB-d1, NB-d2, NB-c1, NB-c2 | FSS-d, FSS-c |
| 6 | SUS, SUD, AWM | NB-d1, NB-d2, NB-c1, NB-c2 | FSS-d, FSS-c |
| 7 | SUS, SUD, RM | NB-d1, NB-d2, NB-c1, NB-c2 | FSS-d, FSS-c |
| 8 | SUS, SUD | NB-d1, NB-d2, NB-c1, NB-c2 | FSS-d, FSS-c, M2S, AWM, RM |
| 9 | SUS, SUD | NB-d1, NB-d2, NB-c1, NB-c2 | FSS-d, FSS-c, M2S, AWM, RM |
| 10 | SUS, SUD | NB-d1, NB-d2, NB-c1, NB-c2 | FSS-d, FSS-c, RM, AWM |
| 11 | SUS, SUD | NB-d1, NB-d2, NB-c1, NB-c2 | FSS-d, FSS-c, RM, M2S |
| 12 | SUS, SUD | NB-d1, NB-d2, NB-c1, NB-c2 | FSS-d, FSS-c, M2S |
| 13 | SUS, SUD | NB-d1, NB-d2, NB-c1, NB-c2 | FSS-d, FSS-c, AWM |
| 14 | SUS, SUD | NB-d1, NB-d2, NB-c1, NB-c2 | FSS-d, FSS-c, RM |
| 15 | SUS, SUD, M2S | NB-d1, NB-d2, NB-c1, NB-c2 | FSS-d, FSS-c, AWM, RM |
| 16 | SUS, SUD, M2S | NB-d1, NB-d2, NB-c1, NB-c2 | FSS-d, FSS-c, AWM |
| 17 | SUS, SUD, M2S | NB-d1, NB-d2, NB-c1, NB-c2 | FSS-d, FSS-c, RM |
| 18 | SUS, SUD, AWM | NB-d1, NB-d2, NB-c1, NB-c2 | FSS-d, FSS-c, M2S, RM |
| 19 | SUS, SUD, AWM | NB-d1, NB-d2, NB-c1, NB-c2 | FSS-d, FSS-c, M2S |
| 20 | SUS, SUD, AWM | NB-d1, NB-d2, NB-c1, NB-c2 | FSS-d, FSS-c, RM |
| 21 | SUS, SUD, RM | NB-d1, NB-d2, NB-c1, NB-c2 | FSS-d, FSS-c, M2s, AWM |
| 22 | SUS, SUD, RM | NB-d1, NB-d2, NB-c1, NB-c2 | FSS-d, FSS-c, M2S |
| 23 | SUS, SUD, RM | NB-d1, NB-d2, NB-c1, NB-c2 | FSS-d, FSS-c, AWM |
| 24 | SUS, SUD, M2s, AWM | NB-d1, NB-d2, NB-c1, NB-c2 | FSS-d, FSS-c, RM |
| 25 | SUS, SUD, RM, M2S | NB-d1, NB-d2, NB-c1, NB-c2 | FSS-d, FSS-c, AWM |
| 26 | SUS, SUD, RM, AWM | NB-d1, NB-d2, NB-c1, NB-c2 | FSS-d, FSS-c, M2S |
| *Note.* SUS = Selective Updating of Sentences task, SUD = Selective Updating of Digits task; NB-d1 = N-Back task with digits level 1; NB-d2 = N-Back task with digits level 2; NB-c1 = N-Back task with colors level 1; NB-c2 = N-Back task with colors level 2; FSS-d = Forward Simple Span task with digits, FSS-c = Forward Simple Span task with colors, M2S = Minus 2 Span task, AWM = Alphabet Working Memory task, RM = Running Memory task. | | | |

| **Supplementary Table 2.** Fit Statistics for Alternative Models of Working Memory (*N* = 106). | | | | | | | |
| --- | --- | --- | --- | --- | --- | --- | --- |
|  |  |  |  |  |  |  |  |
| Model | df | χ^2^ | χ^2^ / *df* | RMSEA | CFI | TLI | SRMR |
| 1 | 41 | 76.079* | 1.856 | .090 | .866 | .821 | .068 |
| 2 | 32 | 62.321* | 1.948 | .095 | .871 | .818 | .067 |
| 3 | 32 | 53.960* | 1.686 | .080 | .883 | .836 | .066 |
| 4 | 32 | 65.942* | 2.060 | .100 | .856 | .797 | .066 |
| 5 | 24 | 52.539* | 2.189 | .106 | .863 | .794 | .064 |
| 6 | 24 | 37.002* | 1.542 | .071 | .919 | .879 | .061 |
| 7 | 24 | 44.277* | 1.845 | .089 | .873 | .810 | .065 |
| 8 | 41 | 61.541* | 1.501 | .069 | .922 | .895 | .070 |
| 9 | 32 | 46.279* | 1.446 | .065 | .939 | .914 | .068 |
| 10 | 32 | 55.735* | 1.741 | .084 | .874 | .823 | .070 |
| 11 | 32 | 47.865* | 1.496 | .068 | .933 | .905 | .061 |
| 12 | 24 | 32.145 | 1.339 | .057 | .961 | .941 | .055 |
| 13 | 24 | 43.231* | 1.801 | .087 | .881 | .821 | .068 |
| 14 | 24 | 40.732* | 1.697 | .081 | .895 | .843 | .064 |
| 15 | 41 | 79.516* | 1.939 | .094 | .853 | .803 | .072 |
| 16 | 32 | 64.689* | 2.022 | .098 | .861 | .804 | .071 |
| 17 | 32 | 67.929* | 2.123 | .103 | .848 | .786 | .067 |
| 18 | 41 | 58.479* | 1.426 | .063 | .933 | .911 | .064 |
| 19 | 32 | 42.817 | 1.338 | .056 | .954 | .935 | .060 |
| 20 | 32 | 50.380* | 1.574 | .074 | .902 | .863 | .066 |
| 21 | 41 | 65.319* | 1.593 | .075 | .907 | .876 | .072 |
| 22 | 32 | 51.134* | 1.598 | .075 | .919 | .886 | .062 |
| 23 | 32 | 60.132* | 1.879 | .091 | .850 | .790 | .071 |
| 24 | 41 | 77.522* | 1.891 | .092 | .861 | .813 | .069 |
| 25 | 41 | 78.382* | 1.912 | .093 | .858 | .809 | .072 |
| 26 | 41 | 60.391* | 1.473 | .067 | .926 | .901 | .063 |
| *Note*. RMSEA = root mean-square error of approximation; CFI = Bentler's comparative fit index; TLI = Tucker-Lewis index; SRMR = standardized root mean-squared residual.  * *p* < .05. | | | | | | | |

| **Supplementary Table 3.** Distribution Statistics for Computerized Tasks Before Exclusion of Outliers; Patients and Controls. | | | | | | | | | | |
| --- | --- | --- | --- | --- | --- | --- | --- | --- | --- | --- |
|  |  |  |  |  |  |  |  |  |  |  |
|  |  |  | PD patients | | | | Healthy controls | | |  |
| Measure | Dependent variable |  | M | SD | skewness (SE) | curtosis (SE) | M | SD | skewness (SE) | curtosis (SE) |
| SUS | percentage of correctly recalled items |  | 57.93 | 17.67 | -.30 (.33) | .16 (.65) | 62.29 | 19.48 | -.1.13 (.33) | 1.32 (.64) |
| SUD | percentage of correctly recalled items |  | 63.97 | 25.76 | -.92 (.33) | .18 (.65) | 76.33 | 19.95 | -1.81 (.33) | 4.05 (.64) |
| NB-d1 | d-prime score |  | 2.33 | .91 | .05 (.33) | -.69 (.65) | 2.55 | 1.14 | -.69 (.33) | -.23 (.64) |
| NB-d2 | d-prime score |  | 1.26 | .65 | -.04 (.33) | -.04 (.65) | 1.16 | .72 | -.03 (.33) | -.11 (.64) |
| NB-c1 | d-prime score |  | 2.66 | .88 | -.55 (.33) | .59 (.65) | 2.70 | .98 | -1.30 (.33) | 1.73 (.64) |
| NB-c2 | d-prime score |  | 1.33 | .69 | .28 (.33) | -.04 (.65) | 1.34 | .78 | -.60 (.33) | 1.15 (.64) |
| FSS-d | sum of correctly recalled items |  | 24.27 | 7.03 | -.73 (.33) | 1.49 (.65) | 26.19 | 7.66 | -.07 (.33) | -.43 (.64) |
| FSS-c | sum of correctly recalled items |  | 22.23 | 5.14 | .93 (.33) | 1.67 (.65) | 23.02 | 7.42 | -.03 (.33) | .29 (.64) |
| M2S | sum of correctly recalled items |  | 40.87 | 13.30 | -.48 (.33) | 1.01 (.65) | 44.39 | 9.96 | .04 (.33) | -.13 (.64) |
| AWM | accuracy / latency |  | 1.66 | .86 | .76 (.33) | 1.36 (.65) | 1.80 | .85 | -.02 (.33) | -.40 (.64) |
| RM | sum of correctly recalled items |  | 19.69 | 7.21 | -.44 (.33) | -.61 (.65) | 18.91 | 8.51 | -.51 (.33) | -.72 (.64) |
| SRT | mean reaction time |  | 396.23 | 41.85 | .77 (.33) | .46 (.65) | 371.32 | 67.93 | 2.03 (.33) | 5.01 (.64) |
| CPT | sum of commission errors |  | 12.29 | 7.52 | 1.47 (.33) | 2.43 (.65) | 8.85 | 4.68 | .52 (.33) | .04 (.64) |
| CPT | sum of omission errors |  | 10.35 | 45.37 | 6.76 (.33) | 47.24 (.65) | 10.59 | 47.19 | 6.02 (.33) | 38.69 (.64) |
| Stroop | interference score |  | 244.00 | 243.97 | -.49 (.33) | -.003 (.65) | 201.81 | 218.56 | -.16 (.35) | -.49 (.68) |
| Sentence recall | percentage of correctly recalled items |  | 59.17 | 14.09 | .13 (.33) | .71 (.65) | 63.31 | 13.22 | -.30 (.33) | .49 (.64) |
| Word list recall | sum of correctly recalled items |  | 18.38 | 4.33 | .44 (.33) | 1.89 (.65) | 20.09 | 3.74 | .34 (.33) | -.36 (.64) |
| *Note*. SUS = Selective Updating of Sentences task, SUD = Selective Updating of Digits task; NB-d1 = N-Back task with digits level 1; NB-d2 = N-Back task with digits level 2; NB-c1 = N-Back task with colors level 1; NB-c2 = N-Back task with colors level 2; FSS-d = Forward Simple Span task with digits, FSS-c = Forward Simple Span task with colors, M2S = Minus 2 Span task; AWM = Alphabet Working Memory task; RM = Running Memory task; SRT = Simple Reaction Time task; CPT = Continuous Performance Task. | | | | | | | | | | |
| *n* = 52 for the PD patients. | | | | | | | | | | |
| *n* = 54 for the healthy controls, except for Stroop (*n* = 47). The missing values for Stroop are due to participants not pressing the response buttons during the task. | | | | | | | | | | |

| **Supplementary Table 4.** Distribution Statistics for Computerized Tasks After Exclusion of Outliers; Patients and Controls. | | | | | | | | | | | | | | | | | | | | | |
| --- | --- | --- | --- | --- | --- | --- | --- | --- | --- | --- | --- | --- | --- | --- | --- | --- | --- | --- | --- | --- | --- |
|  |  | | |  |  | |  | |  | |  | | |  | |  |  | | |  | |
|  |  | | |  | PD patients | | | | | | | | | Healthy controls | | | | | |  | |
| Measure | Dependent variable | | |  | M | | SD | | skewness (SE) | | curtosis (SE) | | | M | | SD | skewness (SE) | | | curtosis (SE) | |
| SUS | percentage of correctly recalled items | | |  | 57.93 | | 17.67 | | -.30 (.33) | | .16 (.65) | | | 62.61 | | 19.52 | -.18 (.33) | | | 1.44 (.64) | |
| SUD | percentage of correctly recalled items | | |  | 63.97 | | 25.76 | | -.92 (.33) | | .18 (.65) | | | 77.77 | | 17.07 | -1.43 (.33) | | | 2.42 (.64) | |
| NB-d1 | d-prime score | | |  | 2.35 | | 0.90 | | .01 (.33) | | -.64 (.66) | | | 2.59 | | 1.14 | -.75 (.33) | | | -.09 (.65) | |
| NB-d2 | d-prime score | | |  | 1.28 | | 0.64 | | -.07 (.33) | | .06 (.66) | | | 1.18 | | 0.72 | -.10 (.33) | | | -.06 (.65) | |
| NB-c1 | d-prime score | | |  | 2.66 | | 0.88 | | -.54 (.33) | | .51 (.66) | | | 2.69 | | 0.99 | -1.27 (.33) | | | 1.64 (.64) | |
| NB-c2 | d-prime score | | |  | 1.33 | | 0.70 | | .28 (.33) | | -.10 (.66) | | | 1.34 | | 0.79 | -.58 (.33) | | | 1.08 (.64) | |
| FSS-d | sum of correctly recalled items | | |  | 24.27 | | 7.03 | | -.73 (.33) | | 1.49 (.65) | | | 26.19 | | 7.66 | -.07 (.33) | | | -.43 (.64) | |
| FSS-c | sum of correctly recalled items | | |  | 22.23 | | 5.14 | | .93 (.33) | | 1.67 (.65) | | | 23.02 | | 7.42 | -.03 (.33) | | | .29 (.64) | |
| M2S | sum of correctly recalled items | | |  | 40.87 | | 13.30 | | -.48 (.33) | | 1.01 (.65) | | | 44.39 | | 9.96 | .04 (.33) | | | -.13 (.64) | |
| AWM | accuracy / latency | | |  | 1.66 | | 0.86 | | .76 (.33) | | 1.36 (.65) | | | 1.80 | | 0.85 | -.02 (.33) | | | -.40 (.64) | |
| RM | sum of correctly recalled items | | |  | 19.69 | | 7.21 | | -.44 (.33) | | -.61 (.65) | | | 18.91 | | 8.51 | -.51 (.33) | | | -.72 (.64) | |
| SRT | mean reaction time | | |  | 396.23 | | 41.85 | | .77 (.33) | | .46 (.65) | | | 358.04 | | 40.54 | .35 (.33) | | | -.58 (.66) | |
| CPT | sum of commission errors | | |  | 12.37 | | 7.57 | | 1.44 (.33) | | 2.33 (.66) | | | 8.85 | | 4.68 | .52 (.33) | | | .04 (.64) | |
| CPT | sum of omission errors | | |  | 2.26 | | 3.64 | | 1.81 (.35) | | 2.31 (.68) | | | 0.48 | | 0.83 | 1.73 (.34) | | | 2.26 (.67) | |
| Stroop | interference score | | |  | 245.03 | | 246.28 | | -.50 (.33) | | -.05 (.66) | | | 201.81 | | 218.56 | -.16 (.35) | | | -.49 (.68) | |
| Sentence recall | percentage of correctly recalled items | | |  | 59.17 | | 14.09 | | .13 (.33) | | .71 (.65) | | | 63.53 | | 13.25 | -.34 (.33) | | | .54 (.64) | |
| Word list recall | sum of correctly recalled items | | |  | 18.38 | | 4.33 | | .44 (.33) | | 1.89 (.65) | | | 20.09 | | 3.74 | .34 (.33) | | | -.36 (.64) | |
| *Note.* SUS = Selective Updating of Sentences task, SUD = Selective Updating of Digits task; NB-d1 = N-Back task with digits level 1; NB-d2 = N-Back task with digits level 2; NB-c1 = N-Back task with colors level 1; NB-c2 = N-Back task with colors level 2; FSS-d = Forward Simple Span task with digits, FSS-c = Forward Simple Span task with colors, M2S = Minus 2 Span task; AWM = Alphabet Working Memory task; RM = Running Memory task; SRT = Simple Reaction Time task; CPT = Continuous Performance Task. | | | | | | | | | | | | | | | | | | | | | |
| *n* = 52 for the PD patients, except for NB-d1 (*n* = 51), NB-d2 (*n* = 51), NB-c1 (*n* = 51), NB-c2 (*n* = 51), CPT commissions (*n* = 51), CPT omissions (*n* = 47), Stroop (*n* = 51). | | | | | | | | | | | | | | | | | | | | | |
| *n* = 54 for the healthy controls, except for SUS (*n* = 53), SUD (*n* = 53), NB-d1 (*n* = 52), NB-d2 (*n* = 52), NB-c1 (*n* = 53), NB-c2 (*n* = 53), SRT ( *n* = 51), CPT omissions (*n* = 48), Stroop (*n* = 47), CCSM (*n* = 53). | | | | | | | | | | | | | | | | | | | | | |
| **Supplementary Table 5.** Descriptive Statistics and Zero-Order Correlations for Measures of Affective Symptoms, Everyday Cognitive Deficits, Global Cognition, and Disease Severity; Patients (*N* = 52). | | | | | | | | | | | | | | | | | | | | | |
|  | |  |  | | |  | |  | |  | |  |  | |  | | |  |  | |  |
| Variable | |  |  | | |  | |  | |  | |  |  | |  | | |  |  | |  |
|  | | 1 | 2 | | | 3 | | 4 | | 5 | | 6 | 7 | | 8 | | | 9 | 10 | |  |
|  | | **Self-reported cognitive symptoms** | | | | | | | | | | | | | | | | | | |  |
| 1. BRIEF-A | | - |  | | |  | |  | |  | |  |  | |  | | |  |  | |  |
| 2. WMQ | | .594* | - | | |  | |  | |  | |  |  | |  | | |  |  | |  |
|  | | **Self-reported affective symptoms** | | | | | | | | | | | | | | | | | | |  |
| 3. GDS-30 | | .420* | .580* | | | - | |  | |  | |  |  | |  | | |  |  | |  |
| 4. LARS | | .087 | .343* | | | .305* | | - | |  | |  |  | |  | | |  |  | |  |
|  | | **Global memory** | | | | | | | | | | | | | | | | | | |  |
| 5. TICS-m | | -.013 | .085 | | | .245 | | -.054 | | - | |  |  | |  | | |  |  | |  |
| 6. TELE | | .054 | .134 | | | .071 | | -.076 | | .347* | | - |  | |  | | |  |  | |  |
|  | | **Disease severity** | | | | | | | | | | | | | | | | | | |  |
| 7. UPDRS part I | | .427* | .481* | | | .387* | | .109 | | -.232 | | -.076 | - | |  | | |  |  | |  |
| 8. UPDRS part II | | .413* | .441* | | | .451* | | .234 | | -.196 | | .033 | .688* | | - | | |  |  | |  |
| 9. PDQ-39 | | .579* | .494* | | | .577* | | .069 | | .014 | | .046 | .667* | | .658* | | | - |  | |  |
| 10. SPDDS | | .495* | .343* | | | .331* | | -.078 | | .046 | | .125 | .547* | | .647* | | | .737* | - | |  |
| *M* | | 57.85 | 48.04 | | | 6.19 | | -27.53 | | 37.17 | | 19.62 | 3.12 | | 9.31 | | | 17.28 | 31.48 | |  |
| *SD* | | 16.57 | 17.13 | | | 5.40 | | 3.97 | | 3.13 | | 0.63 | 2.54 | | 5.29 | | | 11.73 | 6.85 | |  |
| *Note*. BRIEF-A = Behavior Inventory of Executive Functioning; WMQ = Working Memory Questionnaire; GDS-30 = Geriatric Depression Scale-30; LARS = Lille Apathy Rating Scale. TICS-m = Telephone Interview for Cognitive Status Modified; TELE = Telephone Screen Protocol; UPDRS = Unified Parkinson’s Disease Rating Scale; PDQ-39 = Parkinson’s Disease Questionnaire-39; SPDDS = Self-assessment of Parkinson’s Disease Disability Scale. | | | | | | | | | | | | | | | | | | | | |  |
| * *p* < .05. | | | | | | | | | | | | | | | | | | | | |  |

| **Supplementary Table 6.** Zero-Order Correlations for Computerized Tasks; Patients and Controls (*N* = 106). | | | | | | | | | | | | | | | | | |
| --- | --- | --- | --- | --- | --- | --- | --- | --- | --- | --- | --- | --- | --- | --- | --- | --- | --- |
|  |  |  |  |  |  |  |  |  |  |  |  |  |  |  |  |  |  |
| Variable | 1 | 2 | 3 | 4 | 5 | 6 | 7 | 8 | 9 | 10 | 11 | 12 | 13 | 14 | 15 | 16 | 17 |
| 1. SUS | - |  |  |  |  |  |  |  |  |  |  |  |  |  |  |  |  |
| 2. SUD | .583* | - |  |  |  |  |  |  |  |  |  |  |  |  |  |  |  |
| 3. NB-d1 | .240* | .213* | - |  |  |  |  |  |  |  |  |  |  |  |  |  |  |
| 4. NB-d2 | .239* | .302* | .272* | - |  |  |  |  |  |  |  |  |  |  |  |  |  |
| 5. NB-c1 | .158 | .131 | .391* | .265* | - |  |  |  |  |  |  |  |  |  |  |  |  |
| 6. NB-c2 | .253* | .170 | .299* | .502* | .354* | - |  |  |  |  |  |  |  |  |  |  |  |
| 7. FSS-d | .326* | .449* | .048 | 232* | .295* | .114 | - |  |  |  |  |  |  |  |  |  |  |
| 8. FSS-c | .251* | .395* | -.001 | .116 | .091 | .016 | .446* | - |  |  |  |  |  |  |  |  |  |
| 9. M2S | .383* | .641* | .101 | .244* | .144 | .176 | .626* | .517* | - |  |  |  |  |  |  |  |  |
| 10. AWM | .335* | .365* | .230* | .347* | .157 | .143 | .222* | .266* | .347* | - |  |  |  |  |  |  |  |
| 11. RM | .279* | .332* | .058 | .320* | -.014 | .150 | .264* | .402* | .395* | .240* | - |  |  |  |  |  |  |
| 12. SRT | .000 | -.151 | -.283* | -.135 | -.215* | -.259* | -.092 | .013 | -.008 | -.073 | .012 | - |  |  |  |  |  |
| 13. CPT commissions | -.027 | -.066 | -.149 | .013 | -.279* | -.105 | -.315* | -.126 | -.185 | -.116 | .044 | .046 | - |  |  |  |  |
| 14. CPT omissions | -.164 | -.105 | -.221* | -.195 | -.114 | -.224* | -.194 | -.103 | -.047 | -.228* | -.010 | .386* | .292* | - |  |  |  |
| 15. Stroop | .001 | .157 | .061 | .223* | -.026 | .211* | .085 | -.011 | .025 | -.186 | .179 | -.059 | -.029 | -.102 | - |  |  |
| 16. Sentence recall | .670* | .543* | .208* | .164 | .060 | .176 | .344* | .310* | .452* | .261* | .260* | -.151 | -.056 | -.152 | .049 | - |  |
| 17. Word list recall | .140 | .282* | .149 | .195* | .168 | .166 | .196* | .279* | .221* | .131 | .231* | -.223* | -.204* | -.270* | .125 | .276* | - |
| *Note*. SUS = Selective Updating of Sentences task, SUD = Selective Updating of Digits task; NB-d1 = N-Back task with digits level 1; NB-d2 = N-Back task with digits level 2; NB-c1 = N-Back task with colors level 1; NB-c2 = N-Back task with colors level 2; FSS-d = Forward Simple Span task with digits, FSS-c = Forward Simple Span task with colors, M2S = Minus 2 Span task; AWM = Alphabet Working Memory task, RM = Running Memory task; SRT = Simple Reaction Time task; CPT = Continuous Performance Task. | | | | | | | | | | | | | | | | | |
| * *p* < .05. | | | | | | | | | | | | | | | | |  |

| **Supplementary Table 7.** Zero-Order Correlations for Computerized Tasks; Patients (*N* = 52). | | | | | | | | | | | | | | | | | | | | | | | | | | | | | | | | |
| --- | --- | --- | --- | --- | --- | --- | --- | --- | --- | --- | --- | --- | --- | --- | --- | --- | --- | --- | --- | --- | --- | --- | --- | --- | --- | --- | --- | --- | --- | --- | --- | --- |
|  |  | |  | |  | |  | |  | | |  | |  | |  | |  |  | |  |  |  | | |  | |  | |  | |  |
| Variable | 1 | | 2 | | 3 | | 4 | | 5 | | | 6 | | 7 | | 8 | | 9 | 10 | | 11 | 12 | 13 | | | 14 | | 15 | | 16 | | 17 |
| 1. SUS | - | |  | |  | |  | |  | | |  | |  | |  | |  |  | |  |  |  | | |  | |  | |  | |  |
| 2. SUD | .603* | | - | |  | |  | |  | | |  | |  | |  | |  |  | |  |  |  | | |  | |  | |  | |  |
| 3. NB-d1 | .251 | | .205 | | - | |  | |  | | |  | |  | |  | |  |  | |  |  |  | | |  | |  | |  | |  |
| 4. NB-d2 | .350* | | .427* | | .410* | | - | |  | | |  | |  | |  | |  |  | |  |  |  | | |  | |  | |  | |  |
| 5. NB-c1 | .235 | | .125 | | .504* | | .286* | | - | | |  | |  | |  | |  |  | |  |  |  | | |  | |  | |  | |  |
| 6. NB-c2 | .322* | | .319* | | .615* | | .469* | | .682* | | | - | |  | |  | |  |  | |  |  |  | | |  | |  | |  | |  |
| 7. FSS-d | .486* | | .394* | | .177 | | .331* | | .354* | | | .421* | | - | |  | |  |  | |  |  |  | | |  | |  | |  | |  |
| 8. FSS-c | .509* | | .412* | | .011 | | .149 | | .007 | | | .182 | | .316* | | - | |  |  | |  |  |  | | |  | |  | |  | |  |
| 9. M2S | .479* | | .638* | | .250 | | .389* | | .159 | | | .438* | | .586* | | .557* | | - |  | |  |  |  | | |  | |  | |  | |  |
| 10. AWM | .578* | | .524* | | .405* | | .401* | | .421* | | | .415* | | .452* | | .471* | | .516* | - | |  |  |  | | |  | |  | |  | |  |
| 11. RM | .384* | | .330* | | .031 | | .266 | | -.033 | | | .058 | | .298* | | .438* | | .371* | .384* | | - |  |  | | |  | |  | |  | |  |
| 12. SRT | .076 | | .014 | | -.282* | | -.139 | | -.260 | | | -.293* | | -.130 | | .000 | | .070 | -.046 | | .146 | - |  | | |  | |  | |  | |  |
| 13. CPT commissions | -.026 | | .048 | | -.288* | | -.075 | | -.347* | | | -.318* | | -.331* | | -.009 | | -.182 | -.172 | | -.014 | -.001 | - | | |  | |  | |  | |  |
| 14. CPT omissions | -.232 | | -.040 | | -.293* | | -.279 | | -.114 | | | -.282 | | -.300* | | -.130 | | -.014 | -.289* | | -.037 | .356* | .270 | | | - | |  | |  | |  |
| 15. Stroop | -.089 | | .168 | | .020 | | .161 | | -.076 | | | .066 | | .180 | | -.185 | | -.006 | -.253 | | .005 | -.146 | -.133 | | | -.190 | | - | |  | |  |
| 16. Sentence recall | .623* | | .509* | | .251 | | .369* | | .105 | | | .345* | | .418* | | .463* | | .499* | .427* | | .440* | -.139 | -.082 | | | -.192 | | .061 | | - | |  |
| 17. Word list recall | .044 | | .141 | | .250 | | .430* | | .283* | | | .317* | | .162 | | .052 | | .080 | .144 | | .171 | -.112 | -.260 | | | -.309* | | .214 | | .176 | | - |
| *Note.* SUS = Selective Updating of Sentences task, SUD = Selective Updating of Digits task; NB-d1 = N-Back task with digits level 1; NB-d2 = N-Back task with digits level 2; NB-c1 = N-Back task with colors level 1; NB-c2 = N-Back task with colors level 2; FSS-d = Forward Simple Span task with digits, FSS-c = Forward Simple Span task with colors, M2S = Minus 2 Span task; AWM = Alphabet Working Memory task, RM = Running Memory task; SRT = Simple Reaction Time task; CPT = Continuous Performance Task. | | | | | | | | | | | | | | | | | | | | | | | | | | | | | | | | |
| * *p* < .05. | | | | | | | | | | | | | | | | | | | | | | | | | | | | | | | | |
| **Supplementary Table 8.** Zero-Order Correlations for Computerized Tasks; Controls (*N* = 54). | | | | | | | | | | | | | | | | | | | | | | | | | | | | | | | | |
|  | | | | | | | | | | | | | | | | | | | | | | | | | | | | | | | | |
| Variable | | 1 | | 2 | | 3 | | 4 | | 5 | 6 | | 7 | | 8 | | 9 | | 10 | 11 | | 12 | | 13 | 14 | | 15 | | 16 | | 17 | |
| 1. SUS | | - | |  | |  | |  | |  |  | |  | |  | |  | |  |  | |  | |  |  | |  | |  | |  | |
| 2. SUD | | .571* | | - | |  | |  | |  |  | |  | |  | |  | |  |  | |  | |  |  | |  | |  | |  | |
| 3. NB-d1 | | .210 | | .191 | | - | |  | |  |  | |  | |  | |  | |  |  | |  | |  |  | |  | |  | |  | |
| 4. NB-d2 | | .170 | | .223 | | .194 | | - | |  |  | |  | |  | |  | |  |  | |  | |  |  | |  | |  | |  | |
| 5. NB-c1 | | .099 | | .145 | | .310* | | .253 | | - |  | |  | |  | |  | |  |  | |  | |  |  | |  | |  | |  | |
| 6. NB-c2 | | .205 | | .009 | | .080 | | .532* | | .103 | - | |  | |  | |  | |  |  | |  | |  |  | |  | |  | |  | |
| 7. FSS-d | | .175 | | .515* | | -.071 | | .173 | | .249 | -.130 | | - | |  | |  | |  |  | |  | |  |  | |  | |  | |  | |
| 8. FSS-c | | .090 | | .433* | | -.018 | | .103 | | .142 | -.082 | | .527* | | - | |  | |  |  | |  | |  |  | |  | |  | |  | |
| 9. M2S | | .257 | | .622* | | -.079 | | .122 | | .129 | -.103 | | .682* | | .529* | | - | |  |  | |  | |  |  | |  | |  | |  | |
| 10. AWM | | .101 | | .096 | | .089 | | .323* | | -.053 | -.069 | | .002 | | .129 | | .116 | | - |  | |  | |  |  | |  | |  | |  | |
| 11. RM | | .210 | | .429* | | .087 | | .355* | | .000 | .215 | | .256 | | .392* | | .470* | | .132 | - | |  | |  |  | |  | |  | |  | |
| 12. SRT | | .059 | | -.108 | | -.219 | | -.227 | | -.194 | -.269 | | .039 | | .069 | | .034 | | -.010 | -.169 | | - | |  |  | |  | |  | |  | |
| 13. CPT commissions | | .056 | | -.063 | | .069 | | .095 | | -.224 | .171 | | -.274* | | -.250 | | -.097 | | .014 | .092 | | -.232 | | - |  | |  | |  | |  | |
| 14. CPT omissions | | .114 | | .084 | | -.149 | | -.264 | | -.219 | -.401* | | .168 | | -.118 | | .062 | | -.072 | -.073 | | .297* | | .128 | - | |  | |  | |  | |
| 15. Stroop | | .126 | | .250 | | .123 | | .292 | | .051 | .374* | | .020 | | .160 | | .130 | | -.084 | -.357* | | -.075 | | .103 | -.060 | | - | |  | |  | |
| 16. Sentence recall | | .707* | | .570* | | .151 | | -.005 | | .015 | .025 | | .251 | | .204 | | .362* | | .068 | .129 | | -.025 | | .099 | .038 | | .091 | | - | |  | |
| 17. Word list recall | | .194 | | .396* | | .020 | | .001 | | .056 | .024 | | .190 | | .458* | | .371* | | .087 | .325* | | -.219 | | .030 | -.039 | | .064 | | .343* | | - | |
| *Note.* SUS = Selective Updating of Sentences task, SUD = Selective Updating of Digits task; NB-d1 = N-Back task with digits level 1; NB-d2 = N-Back task with digits level 2; NB-c1 = N-Back task with colors level 1; NB-c2 = N-Back task with colors level 2; FSS-d = Forward Simple Span task with digits, FSS-c = Forward Simple Span task with colors, M2S = Minus 2 Span task; AWM = Alphabet Working Memory task, RM = Running Memory task; SRT = Simple Reaction Time task; CPT = Continuous Performance Task. | | | | | | | | | | | | | | | | | | | | | | | | | | | | | | | | |
| * *p* < .05. | | | | | | | | | | | | | | | | | | | | | | | | | | | | | | |  | |

| **Supplementary Table 9.** Standardized Solutions by Confirmatory Factor Analysis for the Selected Working Memory Model (*N* = 106). | | | |
| --- | --- | --- | --- |
|  |  | | |
|  | Factor | | |
| Item | Selective updating | Continuous monitoring | Maintenance of information |
| SUS | .620 |  |  |
| SUD | .945 |  |  |
| NB-d1 |  | .478 |  |
| NB-d2 |  | .667 |  |
| NB-c1 |  | .505 |  |
| NB-c2 |  | .691 |  |
| FSST-d |  |  | .693 |
| FSST-c |  |  | .580 |
| M2S |  |  | .907 |
| *Note.* SUS = Selective Updating of Sentences task, SUD = Selective Updating of Digits task; NB-d1 = N-Back task with digits level 1; NB-d2 = N-Back task with digits level 2; NB-c1 = N-Back task with colors level 1; NB-c2 = N-Back task with colors level 2; FSS-d = Forward Simple Span task with digits, FSS-c = Forward Simple Span task with colors, M2S = Minus 2 Span task. | | | |

| **Supplementary Table 10.** Standard Errors and Confidence Intervals for Standardized Parameter Estimates for the Working Memory Model (*N* = 106). | | | |
| --- | --- | --- | --- |
|  |  |  |  |
| Measure | Est. | S.E. | CI (95 %) |
| SUS | .620 | .124 | .416; .824 |
| SUD | .945 | .098 | .783; 1.106 |
| NB-d1 | .478 | .118 | .283; .673 |
| NB-d2 | .667 | .092 | .515; .819 |
| NB-c1 | .505 | .139 | .276; .734 |
| NB-c2 | .691 | .082 | .556; .827 |
| FSST-d | .693 | .092 | .541; .844 |
| FSST-c | .580 | .071 | .463; .696 |
| M2S | .907 | .059 | .809; 1.004 |
| *Note.* SUS = Selective Updating of Sentences task, SUD = Selective Updating of Digits task; NB-d1 = N-Back task with digits level 1; NB-d2 = N-Back task with digits level 2; NB-c1 = N-Back task with colors level 1; NB-c2 = N-Back task with colors level 2; FSS-d = Forward Simple Span task with digits, FSS-c = Forward Simple Span task with colors, M2S = Minus 2 Span task. | | | |

| **Supplementary Table 11**. Descriptive Statistics and Zero-Order Correlations for Indicator Variables in Model of Working Memory, Affective Symptoms, and Everyday Cognitive Deficits; Patients (*N* = 52). | | | | | | | | | | | | | |
| --- | --- | --- | --- | --- | --- | --- | --- | --- | --- | --- | --- | --- | --- |
|  |  |  |  |  |  |  |  |  |  |  |  |  |  |
| Variable |  |  |  |  |  |  |  |  |  |  |  |  |  |
|  | 1 | 2 | 3 | 4 | 5 | 6 | 7 | 8 | 9 | 10 | 11 | 12 | 13 |
|  | **Working memory task performance** | | | | | | | | | | | | |
| 1. SUS | - |  |  |  |  |  |  |  |  |  |  |  |  |
| 2. SUD | .603* | - |  |  |  |  |  |  |  |  |  |  |  |
| 3. NB-d1 | .263 | .214 | - |  |  |  |  |  |  |  |  |  |  |
| 4. NB-d2 | .368* | .435* | .416* | - |  |  |  |  |  |  |  |  |  |
| 5. NB-c1 | .240 | .132 | .506* | .289* | - |  |  |  |  |  |  |  |  |
| 6. NB-c2 | .338* | .329* | .618* | .476* | .682* | - |  |  |  |  |  |  |  |
| 7. FSS-d | .486* | .394* | .183 | .338* | .355* | .426* | - |  |  |  |  |  |  |
| 8. FSS-c | .509* | .412* | .021 | .161 | .013 | .192 | .316* | - |  |  |  |  |  |
| 9. M2S | .479* | .638* | .263 | .407* | .169 | .451* | .586* | .557* | - |  |  |  |  |
|  | **Self-reported cognitive symptoms** | | | | | | | | | | | | |
| 10. BRIEF-A | .-136 | -.152 | -.141 | -.320* | .-152 | -.230 | -.177 | -.329* | -.261 | - |  |  |  |
| 11. WMQ | .083 | .018 | .122 | -.071 | .025 | .030 | .064 | -.177 | -.028 | .594* | - |  |  |
|  | **Self-reported affective symptoms** | | | | | | | | | | | | |
| 12. GDS | .105 | .081 | .229 | .058 | .072 | .183 | .206 | -.114 | -.040 | .420* | .580* | - |  |
| 13. LARS | .039 | .053 | .107 | -.136 | -.102 | -.028 | .119 | -.052 | -.137 | .089 | .339* | .299* | - |
| *M* | 57.93 | 63.97 | 2.35 | 1.28 | 2.66 | 1.33 | 24.27 | 22.23 | 40.87 | 48.04 | 57.85 | 6.19 | -27.53 |
| *SD* | 17.67 | 25.76 | 0.90 | 0.64 | 0.88 | 0.70 | 7.03 | 5.14 | 13.30 | 17.13 | 16.57 | 5.40 | 3.97 |
| *Note*. SUS = Selective Updating of Sentences task, SUD = Selective Updating of Digits task; NB-d1 = N-Back task with digits level 1; NB-d2 = N-Back task with digits level 2; NB-c1 = N-Back task with colors level 1; NB-c2 = N-Back task with colors level 2; FSS-d = Forward Simple Span task with digits, FSS-c = Forward Simple Span task with colors, M2S = Minus 2 Span task; BRIEF-A = Behavior Inventory of Executive Functioning; WMQ = Working Memory Questionnaire; GDS = Geriatric Depression Scale-30; LARS = Lille Apathy Rating Scale. | | | | | | | | | | | | | |
| * *p* < .05. | | | | | | | | | | | | | |
|  |  |  |  |  |  |  |  |  |  |  |  |  |  |

| **Supplementary Table 12.** Descriptive Statistics and Zero-Order Correlations for Indicator Variables in Model of Working Memory, Affective Symptoms, and Everyday Cognitive Deficits; Controls (*N* = 54). | | | | | | | | | | | | | |
| --- | --- | --- | --- | --- | --- | --- | --- | --- | --- | --- | --- | --- | --- |
|  |  |  |  |  |  |  |  |  |  |  |  |  |  |
| Variable |  |  |  |  |  |  |  |  |  |  |  |  |  |
|  | 1 | 2 | 3 | 4 | 5 | 6 | 7 | 8 | 9 | 10 | 11 | 12 | 13 |
|  | **Working memory task performance** | | | | | | | | | | | | |
| 1. SUS | - |  |  |  |  |  |  |  |  |  |  |  |  |
| 2. SUD | .598* | - |  |  |  |  |  |  |  |  |  |  |  |
| 3. NB-d1 | .216 | .165 | - |  |  |  |  |  |  |  |  |  |  |
| 4. NB-d2 | .131 | .243 | .200 | - |  |  |  |  |  |  |  |  |  |
| 5. NB-c1 | .076 | .171 | .316* | .283 | - |  |  |  |  |  |  |  |  |
| 6. NB-c2 | .176 | -.004 | .078 | .513* | .086 | - |  |  |  |  |  |  |  |
| 7. FSS-d | .137 | .519* | -.091 | .144 | .271 | -.139 | - |  |  |  |  |  |  |
| 8. FSS-c | .076 | .449* | -.051 | .060 | .120 | -.075 | .527* | - |  |  |  |  |  |
| 9. M2S | .264 | .623* | -.097 | .096 | .135 | -.106 | .682* | .529* | - |  |  |  |  |
|  | **Self-reported cognitive symptoms** | | | | | | | | | | | | |
| 10. BRIEF-A | .018 | -.126 | .031 | -.157 | -.184 | .170 | .080 | -.126 | -.130 | - |  |  |  |
| 11. WMQ | .044 | -.075 | .008 | -.188 | -.318* | -.048 | -.044 | -.090 | -.019 | .648* | - |  |  |
|  | **Self-reported affective symptoms** | | | | | | | | | | | | |
| 12. GDS | .121 | .085 | .004 | -.344* | -.118 | -.219 | .162 | -.022 | -.085 | .645* | .439* | - |  |
| 13. LARS | .056 | .021 | .171 | -.233 | -.141 | .-113 | -.082 | .002 | .067 | .225 | .213 | .336* | - |
| *M* | 62.61 | 77.77 | 2.59 | 1.18 | 2.69 | 1.34 | 26.19 | 23.02 | 44.39 | 40.00 | 48.85 | 2.72 | -28.06 |
| *SD* | 19.52 | 17.07 | 1.14 | 0.72 | 0.99 | 0.79 | 7.66 | 7.42 | 9.96 | 18.65 | 12.57 | 3.34 | 3.71 |
| *Note*. SUS = Selective Updating of Sentences task, SUD = Selective Updating of Digits task; NB-d1 = N-Back task with digits level 1; NB-d2 = N-Back task with digits level 2; NB-c1 = N-Back task with colors level 1; NB-c2 = N-Back task with colors level 2; FSS-d = Forward Simple Span task with digits, FSS-c = Forward Simple Span task with colors, M2S = Minus 2 Span task; BRIEF-A = Behavior Inventory of Executive Functioning; WMQ = Working Memory Questionnaire; GDS = Geriatric Depression Scale-30; LARS = Lille Apathy Rating Scale. | | | | | | | | | | | | | |
| * *p* < .05. | | | | | | | | | | | | | |

| **Supplementary Table 13.** Standard Errors and Confidence Intervals for Standardized Parameter Estimates for the Model of Working Memory, Affective Symptoms, and Everyday Cognitive Deficits (*N* = 106). | | | |
| --- | --- | --- | --- |
|  |  |  |  |
| Measure | Est. | S.E. | CI (95 %) |
| **Working memory task performance** |  |  |  |
| SUS | .608 | .126 | .401; .816 |
| SUD | .960 | .099 | .797; 1.123 |
| NB-d1 | .461 | .131 | .246; .676 |
| NB-d2 | .696 | .114 | .509; .884 |
| NB-c1 | .500 | .148 | .256; .744 |
| NB-c2 | .677 | .086 | .536; .818 |
| FSST-d | .694 | .093 | .541; .847 |
| FSST-c | .583 | .070 | .467; .698 |
| M2S | .904 | .059 | .808; 1.001 |
| **Self-reported cognitive symptoms** |  |  |  |
| WMQ | .814 | .069 | .701; .928 |
| BRIEF | .777 | .071 | .660; .894 |
| **Self-reported affective symptoms** |  |  |  |
| GDS-30 | .896 | .111 | .713; 1.078 |
| LARS | .365 | .107 | .189; .540 |
| *Note.* SUS = Selective Updating of Sentences task, SUD = Selective Updating of Digits task; NB-d1 = N-Back task with digits level 1; NB-d2 = N-Back task with digits level 2; NB-c1 = N-Back task with colors level 1; NB-c2 = N-Back task with colors level 2; FSS-d = Forward Simple Span task with digits, FSS-c = Forward Simple Span task with colors, M2S = Minus 2 Span task;WMQ = Working Memory Questionnaire; BRIEF-A = Behavior Rating Inventory of Executive Function; GDS-30 = Geriatric Depression Scale-30; LARS = Lille Apathy Rating Scale. | | | |

| **Supplementary Table 14.** Descriptive Statistics and Zero-Order Correlations for Measures of Working Memory and Global Cognition; Patients (*N* = 52). | | | | | | | | | | | |
| --- | --- | --- | --- | --- | --- | --- | --- | --- | --- | --- | --- |
|  |  |  |  |  |  |  |  |  |  |  |  |
| Variable |  |  |  |  |  |  |  |  |  |  |  |
|  | 1 | 2 | 3 | 4 | 5 | 6 | 7 | 8 | 9 | 10 | 11 |
|  | **Working memory task performance** | | | | | | | | | | |
| 1. SUS | - |  |  |  |  |  |  |  |  |  |  |
| 2. SUD | .603* | - |  |  |  |  |  |  |  |  |  |
| 3. NB-d1 | .269 | .217 | - |  |  |  |  |  |  |  |  |
| 4. NB-d2 | .362* | .433* | .417* | - |  |  |  |  |  |  |  |
| 5. NB-c1 | .239 | .131 | .505* | .289* | - |  |  |  |  |  |  |
| 6. NB-c2 | .338* | .329* | .621* | .475* | .682* | - |  |  |  |  |  |
| 7. FSS-d | .486* | .394* | .185 | .337* | .354* | .426* | - |  |  |  |  |
| 8. FSS-c | .509* | .412* | .024 | .158 | .012 | .192 | .316* | - |  |  |  |
| 9. M2S | .479* | .638* | .272* | .401* | .167 | .451* | .586* | .557* | - |  |  |
|  | **Global cognition** | | | | | | | | | | |
| 10. TICS-m | .313* | .255 | .305* | .354* | .255 | .310* | .355* | .149 | .147 | - |  |
| 11. TELE | .275* | .214 | .283* | .139 | .161 | .218 | .218 | .197 | .167 | .347* | - |
| *M* | 57.93 | 63.97 | 2.35 | 1.28 | 2.66 | 1.33 | 24.27 | 22.23 | 40.87 | 37.17 | 19.62 |
| *SD* | 17.67 | 25.76 | 0.90 | 0.64 | 0.88 | 0.70 | 7.03 | 5.14 | 13.30 | 3.13 | 0.63 |
| *Note.* SUS = Selective Updating of Sentences task, SUD = Selective Updating of Digits task; NB-d1 = N-Back task with digits level 1; NB-d2 = N-Back task with digits level 2; NB-c1 = N-Back task with colors level 1; NB-c2 = N-Back task with colors level 2; FSS-d = Forward Simple Span task with digits, FSS-c = Forward Simple Span task with colors, M2S = Minus 2 Span task, TICS-m = Telephone Interview for Cognitive Status Modified; TELE = Telephone Screening Protocol. | | | | | | | | | | | |
| * *p* < .05. | | | | | | | | | | | |

| **Supplementary Table 15.** Descriptive Statistics and Zero-Order Correlations for Measures of Working Memory and Disease Severity; Patients (*N* = 52). | | | | | | | | | | | | | |
| --- | --- | --- | --- | --- | --- | --- | --- | --- | --- | --- | --- | --- | --- |
|  |  |  |  |  |  |  |  |  |  |  |  |  |  |
| Variable |  |  |  |  |  |  |  |  |  |  |  |  |  |
|  | 1 | 2 | 3 | 4 | 5 | 6 | 7 | 8 | 9 | 10 | 11 | 12 | 13 |
|  | **Working memory task performance** | | | | | | | | | | | | |
| 1. SUS | - |  |  |  |  |  |  |  |  |  |  |  |  |
| 2. SUD | .603* | - |  |  |  |  |  |  |  |  |  |  |  |
| 3. NB-d1 | .269 | .217 | - |  |  |  |  |  |  |  |  |  |  |
| 4. NB-d2 | .368* | .435* | .420* | - |  |  |  |  |  |  |  |  |  |
| 5. NB-c1 | .240 | .131 | .507* | .291* | - |  |  |  |  |  |  |  |  |
| 6. NB-c2 | .340* | .330* | .621* | .478* | .683* | - |  |  |  |  |  |  |  |
| 7. FSS-d | .486* | .394* | .185 | .338* | .355* | .426* | - |  |  |  |  |  |  |
| 8. FSS-c | .509* | .412* | .024 | .161 | .013 | .193 | .316* | - |  |  |  |  |  |
| 9. M2S | .479* | .638* | .271 | .408* | .168 | .453* | .586* | .557* | - |  |  |  |  |
|  | **Disease severity** | | | | | | | | | | | | |
| 10. UPDRS part I | -.167 | -.147 | -.080 | -.217 | .011 | -.099 | -.101 | -.262 | -.106 | - |  |  |  |
| 11. UPDRS part II | -.252 | -.213 | -.030 | -.259 | -.075 | -.082 | -.008 | -.294* | -.133 | .688* | - |  |  |
| 12. PDQ-39 | -.185 | -.161 | .005 | -.072 | -.117 | -.008 | .006 | -.288* | -.113 | .667* | .658* | - |  |
| 13. SPDDS | -.117 | -.149 | .047 | -.054 | -.023 | -.051 | .076 | -.325* | -.014 | .547* | .647* | .737* | - |
| *M* | 57.93 | 63.97 | 2.35 | 1.28 | 2.66 | 1.33 | 24.27 | 22.23 | 40.87 | 3.12 | 9.31 | 17.28 | 31.48 |
| *SD* | 17.67 | 25.76 | 0.90 | 0.64 | 0.88 | 0.70 | 7.03 | 5.14 | 13.30 | 2.54 | 5.29 | 11.73 | 6.85 |
| *Note.* SUS = Selective Updating of Sentences task, SUD = Selective Updating of Digits task; NB-d1 = N-Back task with digits level 1; NB-d2 = N-Back task with digits level 2; NB-c1 = N-Back task with colors level 1; NB-c2 = N-Back task with colors level 2; FSS-d = Forward Simple Span task with digits, FSS-c = Forward Simple Span task with colors, M2S = Minus 2 Span task, UPDRS = Unified Parkinson’s Disease Rating Scale; PDQ-39 = Parkinson’s Disease Questionnaire-39; SPDDS = Self-assessment of Parkinson’s Disease Disability Scale. | | | | | | | | | | | | | |
| * *p* < .05. | | | | | | | | | | | | | |
|  | | | | | | | | | | | | |  |
